# A risk-stratified framework for in-house PLA 3D printing of non-critical medical device spare parts: a cost-effectiveness analysis

**DOI:** 10.3389/fmed.2026.1911165

**Published:** 2026-08-27

**Authors:** Kun Li, Xinghua Ma, Lei Su, Yinyan Sun, Qian Mu, Dingmeng Luo, Jing Cheng

**Affiliations:** Department of Medical Equipment, 3201 Hospital of Xi’an Jiaotong University Health Science Center, Hanzhong, Shaanxi, China

**Keywords:** 3D printing, cost-Benefit analysis, medical device maintenance, non-critical spare parts, risk classification, supply chain resilience

## Abstract

**Background:**

Procurement of non-critical medical device spare parts faces major challenges. These include high costs, long lead times, and supply chain fragility. In-house additive manufacturing (AM), specifically Polylactic Acid (PLA) Fused Deposition Modeling (FDM), offers a potential solution but lacks systematic safety and economic validation.

**Objective:**

This study aimed to establish a risk-stratified framework for identifying suitable non-critical spare parts and to evaluate the engineering feasibility, economic impact, and operational implications of in-house PLA FDM 3D printing.

**Methods:**

A three-dimensional risk classification matrix (Functional, Contact, Load; F-C-L) was developed to screen eligible parts (F0, L0, C ≤ C1). Forty-two part models were selected. Engineering evaluations were conducted. These assessed dimensional accuracy, assembly fit, and simulated reliability. A comprehensive economic analysis was performed. It compared in-house printing with traditional OEM procurement. Metrics included unit cost, lead time, downtime, and estimated benefits.

**Results:**

The mean dimensional error was 0.18 ± 0.07 mm. All deviations remained within the ±0.5 mm tolerance. The first-print installation success rate was 92.9%, reaching 100% after minor adjustments. User-perceived performance was rated as comparable or acceptable. All parts passed scenario-specific durability tests (e.g., 20,000 press cycles for buttons). Economically, unit costs dropped significantly. 3D-printed parts cost CNY 66.86 vs. CNY 530.92 for OEM parts (*P* < 0.001), an 87.4% reduction. Supply lead time fell drastically from 202.8 h to 11.9 h (*P* < 0.001). This yielded a 94.1% reduction in annual downtime for the 20 representative parts analyzed. The estimated annual economic benefit was approximately CNY 2.66 million. The return on investment was high. Sensitivity analyses confirmed the robustness of these cost advantages.

**Conclusion:**

Within clearly defined safety boundaries, in-house PLA FDM 3D printing is technically viable. It is also economically advantageous. The proposed F-C-L risk matrix provides a pragmatic framework. It enhances hospital supply chain resilience and operational efficiency.

## 1 Introduction

Modern hospitals depend significantly on large-scale medical equipment and complex therapeutic systems (1). As the installed base and sophistication of this equipment continue to grow, challenges such as equipment downtime, degraded operation, and substantial repair costs due to failures of non-critical spare parts—including enclosure components, control and actuation parts, support elements, and cable-management accessories—have become persistent issues in medical equipment management (2). On one hand, original equipment manufacturer (OEM) spare parts for imported devices are often costly and come with long lead times; for certain discontinued models, spare parts may even be “priced but unavailable.” (3) On the other hand, while non-critical structural parts do not directly impact core measurement or therapeutic functions, they often require replacement due to cosmetic defects, impaired operability, or potential mechanical safety hazards; failure to address these issues may compromise care quality and patient safety.

From a public health and health-system operations perspective, medical equipment availability and reliability are fundamental to service continuity (4). Non-critical structural or auxiliary component failures can disable devices or force downgraded operation, even when core functions remain intact. This results in reduced throughput, prolonged patient wait times, and increased clinician and technician workloads (5). In high-volume tertiary hospitals, the cumulative effect of such “unplanned downtime” on service efficiency and resource allocation is significant.

3D printing, also known as additive manufacturing (AM) (6, 7), presents several advantages, including on-demand production, a low marginal cost for complex geometries, and the potential to shorten supply chains (8, 9). It has seen widespread adoption in various medical applications (10, 11), such as anatomical models, surgical guides, patient-specific implants, and rehabilitation assistive devices (12–15). A systematic review conducted by Rouf et al. (16) highlighted that the mechanical and tribological performance of polymer 3D-printed components is significantly influenced by the choice of material systems and process parameters. Proper optimization of factors such as layer thickness, infill density, raster angle, and temperature can substantially enhance the tensile strength, fatigue resistance, and wear performance of thermoplastics like PLA (17, 18).

Within the medical equipment domain, initial feasibility has been demonstrated, such as Cai (19) utilizing PLA-FDM to fabricate syringe clamps for infusion pumps, offering a rapid alternative to OEM parts. The COVID-19 pandemic further showcased 3D printing's rapid-response capability in addressing equipment shortages and bolstering supply chain resilience (20, 21). However, while recent reviews on broader medical AM applications (22–25) highlight broad advances in surgical planning and bioscaffolds, they also underscore persistent gaps in regulatory standardization and comprehensive cost-benefit analyses for routine hospital maintenance. This gap—between ad-hoc emergency use and systematic, economically justified integration—motivates the present study.

From a supply chain and maintenance perspective, a case analysis by Chaldoupis et al. (26) across various medical device companies indicated that extending additive manufacturing (AM) from “custom parts” to the production of “standard/common parts” could yield cumulative multidimensional performance improvements—such as reduced delivery times and lower inventory and transportation costs—through a build-to-model approach, digital kitting, and shared capabilities. In China, studies on spare parts at the equipment level have predominantly concentrated on case-based practices and experience-driven economic comparisons (27). However, empirical research that systematically integrates engineering performance testing, maintenance workflows, and cost-effectiveness analysis remains scarce.

PLA is a bio-based thermoplastic characterized by stable printability, low warpage, good dimensional repeatability, and relatively low material costs, making it widely used in Fused Deposition Modeling (FDM) 3D printing (28, 29). Although its limited thermal resistance, interlayer anisotropy, and sensitivity to high-temperature sterilization restrict its application to appropriate scenarios. It offers practical advantages for non-critical structural components that do not require high-temperature resistance, high corrosion resistance, or high load-bearing capacity, and can be cleaned through wiping or low-temperature disinfection (30, 31).

In contrast to critical functional components, non-critical spare parts typically do not incorporate complex electronics or require precision calibration. Their structures primarily comprise plastic housings, supports, and connectors. Manufacturing processes for these parts remain relatively straightforward (32). Defining this category's boundaries is essential. Non-critical parts are defined as those whose failure does not directly alter therapeutic output or cause physical harm. However, such failures may still impede operability or create secondary safety risks. Components involving electrical insulation, patient-contact surfaces, radiation shielding, or primary load-bearing functions are strictly excluded. Current equipment management frameworks often group these parts with critical components. This enforces uniform OEM procurement and maintenance protocols. Consequently, flexibility in pricing, lead times, and inventory control remains limited. Therefore, establishing a differentiated technical substitution pathway for non-critical spare parts is both engineering-feasible and managerially significant.

Building on prior reports, this study aims to address three primary questions: First, under what risk stratification and usage assumptions can PLA-based FDM 3D printing safely and reliably substitute for specific types of non-critical spare parts in medical equipment, within an explicit risk framework? Second, while meeting basic functional and reliability requirements, how much improvement can PLA 3D-printed spare parts provide—relative to OEM procurement—in terms of cost, lead time, and equipment downtime? Third, how can an evaluation framework be constructed that jointly considers engineering performance, clinical use, and economic value to support hospital-level decision-making for spare parts management and the development of in-house 3D printing platforms?

Accordingly, from the perspective of medical equipment management, and recognizing the material constraints and risk boundaries discussed above, this study propose a risk stratification scheme for non-critical spare parts along with inclusion and exclusion criteria, providing illustrative substitution ranges across multiple device types. Drawing on the polymer 3D-printing mechanical testing methods summarized by Rouf et al. (16), this study develop a hospital-oriented, integrated engineering evaluation workflow that spans dimensional accuracy, assembly/fit, functional validation, and service-life assessment. Leveraging domestic repair case studies and international supply-chain research, this study establish a cost-effectiveness-centered economic analysis framework and embed paired before-and-after data from 20 representative spare parts to systematically compare an OEM procurement model with an in-house 3D printing model across four dimensions: total cost per part, spare parts lead time, equipment downtime, and economic benefits.

Our main contributions are as follows: (1) a structured, risk-based framework for the in-hospital replacement of non-critical medical device spare parts; (2) an integrated engineering-economic evaluation pipeline that combines dimensional accuracy, functional validation, and cost-effectiveness; and (3) real-world paired before-and-after economic evidence from a tertiary hospital. In the Discussion section, this study contextualize our findings within the broader literature on 3D printing in healthcare, considering material-process factors and sustainability. Additionally, this study propose implementation pathways and practical considerations specifically tailored for tertiary hospitals in China.

## 2 Materials and methods

### 2.1 Study design and setting

This study employed a combined design that included (i) engineering experiments and (ii) a single-center, historical before–after economic evaluation. For the engineering component, this study assessed representative non-critical spare parts to ascertain whether PLA-based FDM 3D-printed substitutes achieved satisfactory dimensional accuracy, assembly fit, and in-use reliability. For the economic component, this study analyzed equipment operation and maintenance records from the same hospital both before and after the implementation of an in-house 3D-printing program. Out of 42 eligible spare-part types, this study selected 20 representative models for paired before–after comparisons.

**Figure 1:**
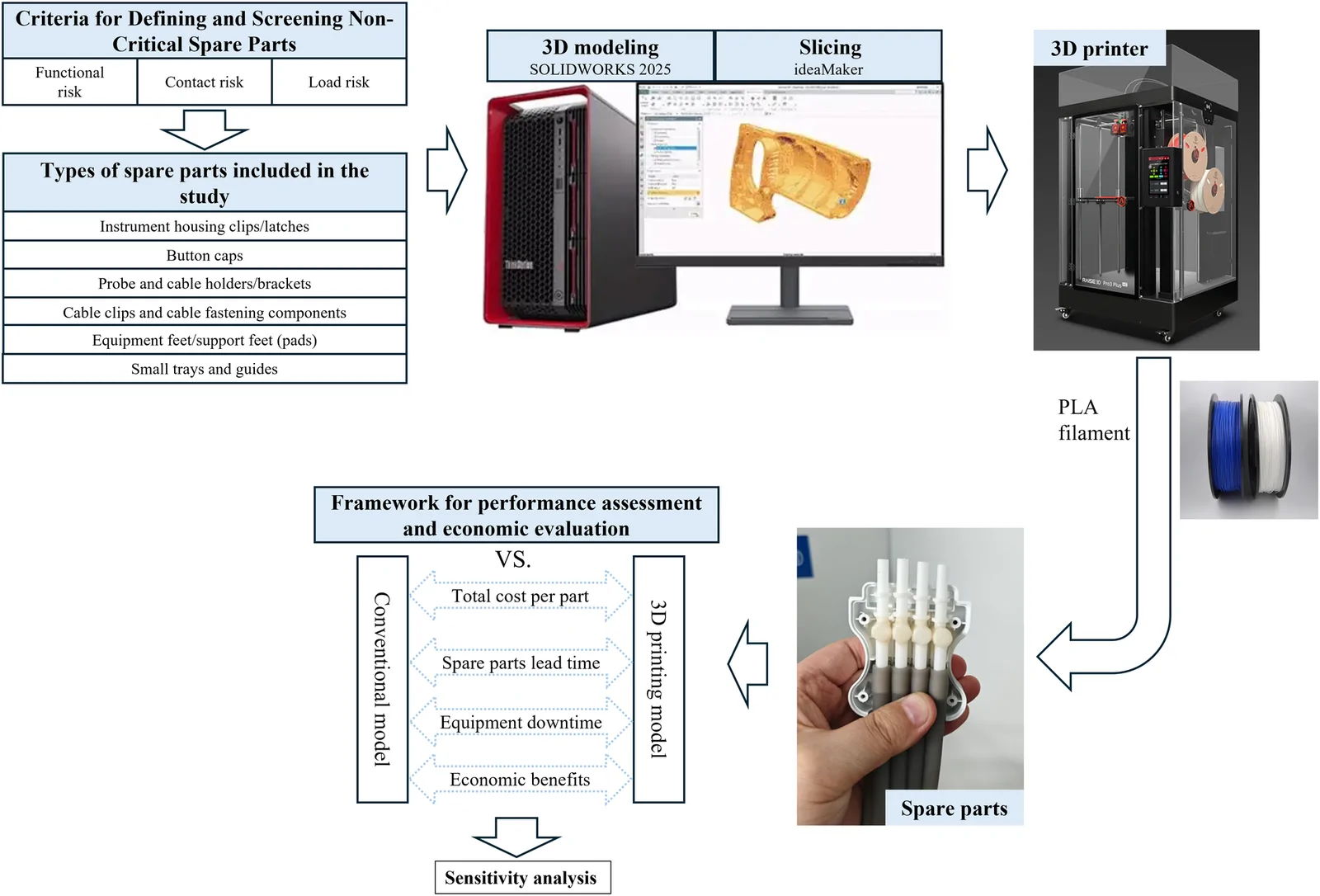
Overview of the study design and workflow.

The study was conducted within the Medical Equipment Department of a tertiary comprehensive hospital, which operates over 5,000 devices, including imaging, laboratory, monitoring, infusion, and specialty equipment. The hospital maintains comprehensive asset ledgers and utilizes a computerized maintenance management/information system. To clearly present the overall study roadmap, this study developed the following study workflow framework diagram, as illustrated in Figure 1.

### 2.2 Operational definition and screening of “non-critical spare parts”

To ensure safety and alignment with medical device regulatory principles, this study developed an operational definition of non-critical spare parts, together with explicit inclusion and exclusion criteria. This definition is grounded in the Regulations on the Supervision and Administration of Medical Devices(China) (33), ISO 14971:2019, and internal hospital quality and safety standards. While existing medical device risk management standards such as ISO 14971:2019 provide a foundation for system-level hazard identification, they do not offer a granular, component-level screening tool tailored to the operational realities of in-house 3D printing for spare part replacement. To address this gap, the proposed F-C-L matrix translates regulatory principles into a binary-decision framework that enables clinical engineers to rapidly identify printable non-critical parts without formal risk-management training. Given the substantial differences in functional importance, use scenarios, and safety risks associated with spare parts, this study adopted a risk-oriented screening strategy to prevent the indiscriminate application of 3D printing across all spare part categories. The scope of the study was limited to non-critical structural parts that (i) do not directly participate in core diagnostic or therapeutic functions and (ii) whose failure would not directly impact patient safety or clinical outcomes. To enhance transparency, consistency, and reproducibility, this study established a standardized, multidimensional risk stratification method.

#### 2.2.1 Risk classification matrix and eligibility rules

To systematically identify non-critical spare parts suitable for in-hospital 3D-printed replacement, this study constructed a risk classification matrix comprising three independent dimensions: functional risk (F), patient contact risk (C), and mechanical load risk (L). This framework is grounded in medical device risk-management principles and engineering safety assessment methods.

(1) Functional risk (F) assesses the role of the part within the device's functional architecture and the consequences of failure. The classification includes: F0 (non-functional part), which does not participate in core diagnostic or therapeutic functions; failure does not directly affect key performance metrics or clinical outcomes (e.g., decorative covers, button caps, label frames); F1 (auxiliary functional part), which supports auxiliary functions; failure may indirectly impact usability or stability but does not directly alter core functional outputs; and F2 (critical functional part), which is directly involved in diagnosis, therapy, or energy transfer; failure may result in malfunction and clinical risk. (2) Patient contact risk (C) evaluates the degree and duration of direct or indirect patient contact during normal use. The categories are: C0 (no patient contact), indicating no direct or indirect contact (e.g., external housings, internal structural supports); C1 (transient or indirect contact), where brief or indirect contact may occur during operation but is neither long-term adherent nor invasive; and C2 (direct/long-term contact), which involves direct, prolonged, or invasive contact during device operation.

(3) Mechanical load risk (L) assesses mechanical loading and its implications for structural integrity and safety. The classification includes: L0 (low load), indicating none or minimal load, primarily used for positioning, covering, or light support; L1 (moderate load), which experiences mechanical stress or repetitive operational loads, where failure may affect stability or service life; and L2 (high load), indicating high static or dynamic load, or involvement in energy transfer, precision motion, or safety-related structures.

**Figure 2:**
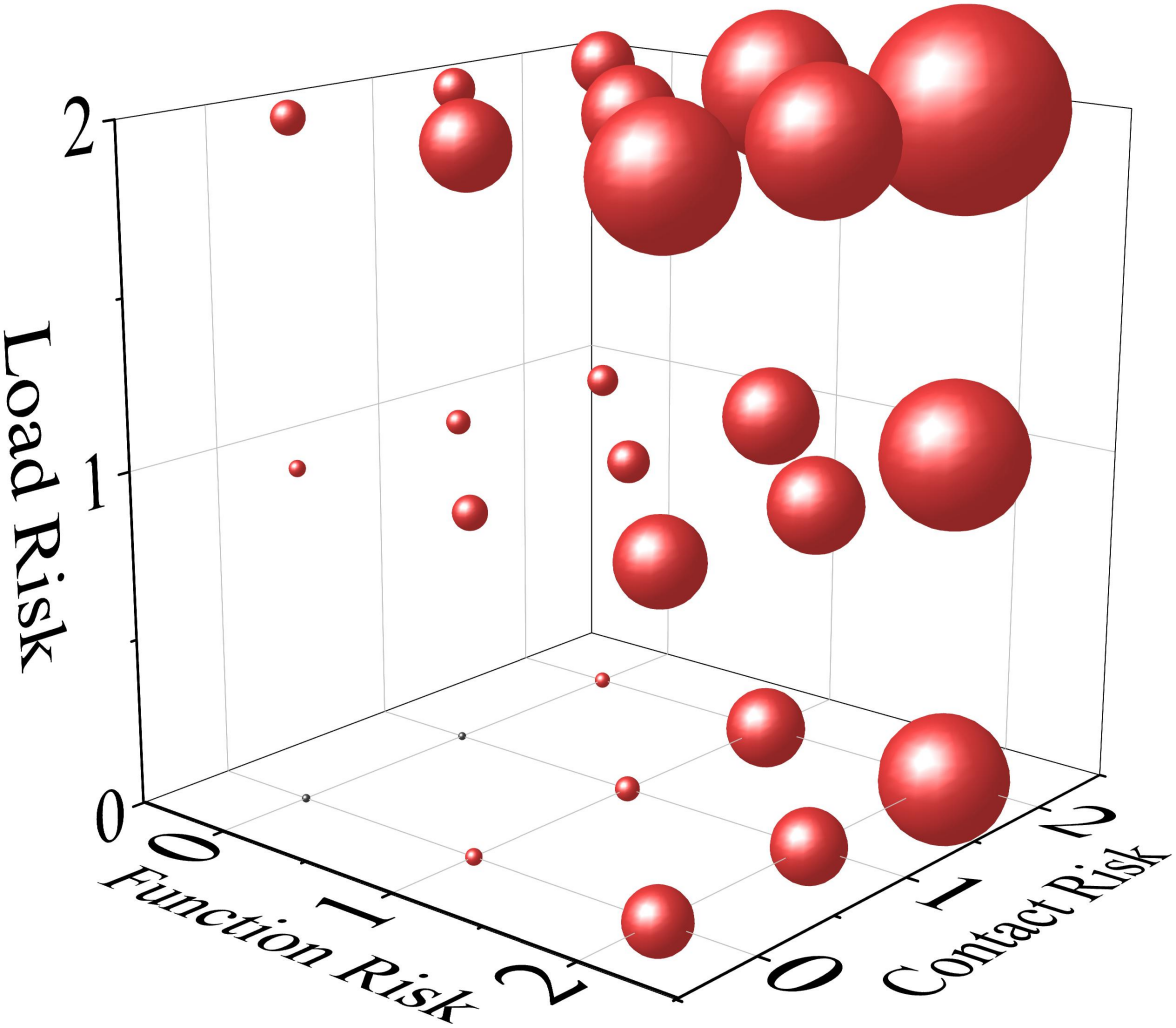
Three-dimensional risk classification matrix.

Each spare part was coded as a combination of F, C, and L. Only parts meeting the criteria of F0, L0, and C ≤ C1—specifically, F0–C0–L0 or F0–C1–L0—were eligible for PLA FDM replacement. As illustrated by the two black dots in Figure 2, the red dot indicates an unacceptable risk point. The following components were explicitly excluded: any F1 or F2 component; any L1 or L2 component; any C2 component (direct, prolonged, or invasive patient contact); and parts involving electrical safety, energy output, fluid pathways, or precision calibration.

Using this matrix, we screened common hospital equipment spare parts and identified 42 eligible non-critical part types. Typical eligible items included button caps, decorative housing components, cable clips, support brackets, and protective covers. This standardized eligibility rule set provided a reproducible basis for subsequent modeling, process selection, engineering testing, and economic evaluation.

To ensure reproducible application of the F-C-L matrix, the following stepwise procedure was used for each candidate spare part:

Step 1 (Functional assessment—F): Consult the device's service manual and the hospital's equipment incident database to determine whether the part appears in any safety-critical or performance-critical component list. If the part is absent from these lists and its failure does not trigger device alarms or affect calibrated outputs, it is coded F0. If the part supports auxiliary functions (e.g., display brackets, cable organizers) but its failure could indirectly affect device stability, it is coded F1. If the part is directly involved in diagnosis, therapy, or energy transfer, it is coded F2 and automatically excluded.
Step 2 (Contact assessment—C): Determine the nature and duration of patient contact during normal clinical use, based on the device's intended-use description and observation of actual clinical workflows. Parts with no patient contact path are coded C0. Parts with intermittent, indirect contact (e.g., a button cap touched briefly by a patient's finger during a procedure) are coded C1. Parts with direct, prolonged, or invasive contact are coded C2 and automatically excluded.
Step 3 (Load assessment—L): Estimate the mechanical loading based on the part's location, mounting method, and function within the device, cross-referenced with the device's mechanical assembly drawings where available. Parts used solely for positioning, covering, or cosmetic purposes with no transmitted force are coded L0. Parts subject to measurable operational stress (e.g., load-bearing arms, gear teeth, sliding rails) are coded L1 or L2 and automatically excluded.
Step 4 (Final eligibility determination): Only parts receiving the combination F0–C0/C1–L0 proceed to 3D printing. All others are rejected.

This procedure was applied independently by two senior clinical engineers to a random subset of 10 spare parts, yielding 100% inter-rater agreement (*κ* = 1.0), confirming the reproducibility of the classification scheme.

#### 2.2.2 Inclusion criteria

1. External structural or auxiliary parts (e.g., plastic housing clips, decorative panels, button caps, probe/cable holders, cable-management clips, support feet/pads, small trays/guides).
2. Components not directly involved in measurement, therapy, or life-support functions; these parts do not alter calibrated performance parameters.
3. Parts that have no more than transient or indirect contact with patients (C1); they should be amenable to routine wiping or low-temperature disinfection and must not enter sterile lumens or the surgical field.
4. Failures that mainly manifest as cosmetic defects, impaired operability, or potential mechanical safety hazards, highlighting a clear clinical need for replacement.
5. Records of recurrent failures or replacements within the past three years.

#### 2.2.3 Exclusion criteria

1. Parts associated with electrical safety, shielding/protection, or high-energy transfer (e.g., high-voltage connectors, protective doors on x-ray/radiotherapy devices, radiation shielding panels).
2. Critical structural components necessary for precision transmission or high-load bearing (e.g., gearbox gears, motion rails, brakes, load-bearing support arms).
3. Locations subjected to long-term exposure to pharmaceuticals or body fluids that necessitate high-temperature steam sterilization or strongly corrosive chemical disinfection.
4. Key components for which regulations, standards, or OEM instructions explicitly prohibit non-OEM replacements.

This risk stratification does not rely solely on part size or price; instead, it integrates potential impacts on core device functionality, patient safety, and regulatory compliance. By excluding load-bearing, high-energy, protective or shielding components, as well as parts intended for long-term contact with body fluids, this approach aims to significantly mitigate safety risks associated with the substitution of 3D-printed components, thereby providing a feasible operational pathway for hospitals to explore additive manufacturing within a compliance-oriented framework.

#### 2.2.4 Screening procedure and part selection

**Figure 3:**
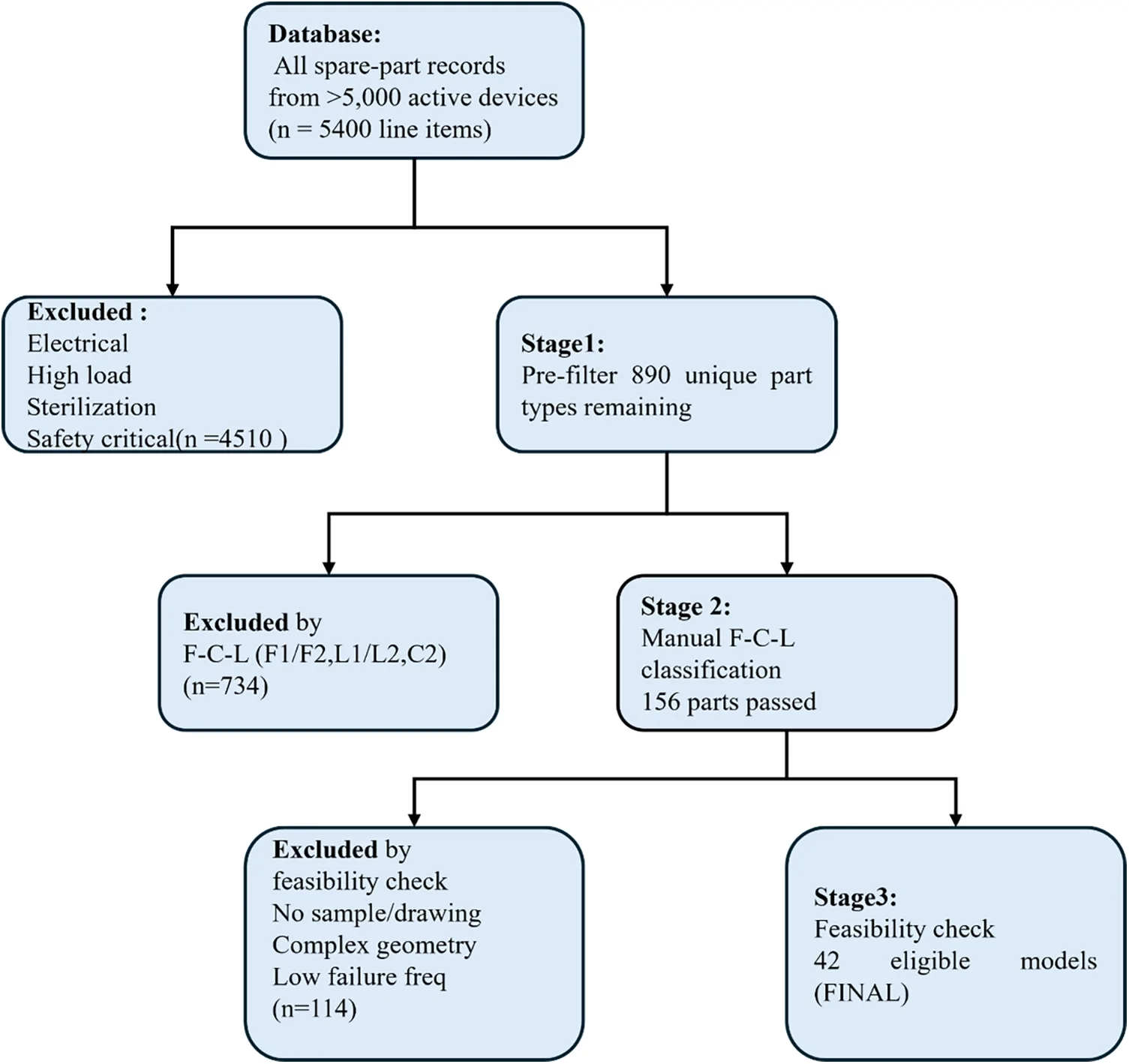
PRISMA-style flow diagram of the three-stage screening procedure for non-critical medical device spare parts.

The identification of the 42 eligible spare-part models followed a three-stage screening procedure applied to the hospital's medical devices maintenance management system database, which contained records of all spare-part procurements and equipment failures over the preceding three years (Figure 3).

Stage 1 (Automated pre-filter): All spare parts associated with the hospital's active device fleet (>5,000 units) were retrieved from the database. Parts were excluded if their procurement records indicated they belonged to any of the following categories: (i) electrical components (connectors, circuit boards, sensors); (ii) high-load mechanical components (gears, bearings, rails, braking mechanisms); (iii) parts requiring high-temperature sterilization or fluid-path contact; (iv) parts explicitly designated as “safety-critical” in the device's service manual. This pre-filter reduced the candidate pool from several thousand line items to 890 unique part types.

Stage 2 (Manual F-C-L classification): Two senior clinical engineers independently reviewed the remaining 890 part types. For each part, they applied the three-step F-C-L classification procedure described in Section 2.2.1 (consulting service manuals, observing clinical workflows, and reviewing mechanical drawings). Inter-rater agreement was assessed on a random subset of 30 parts (Cohen's *κ* = 0.93). Discrepancies were resolved by consensus discussion with a third engineer. Parts receiving F0–C0/C1–L0 codes proceeded to the next stage, yielding 156 candidates.

Stage 3 (Practical feasibility check): The 156 parts that passed Stage 2 were further evaluated for 3D printing feasibility based on: (i) availability of a physical sample or OEM technical drawing for reverse modeling; (ii) absence of complex internal geometries requiring soluble supports that could not be reliably removed in a hospital workshop setting; (iii) historical failure frequency ≥1 incident in the past three years (to ensure clinical relevance). This final filter yielded the 42 eligible models.

The final eligible set comprises six categories, totaling 42 models as follows: housing clips and fasteners (12 models), operator-interface parts (8 models), probe and cable holders (7 models), cable-management clips and fixation parts (6 models), device feet, pads, and support feet (5 models), and small auxiliary trays and guides (4 models) — for example, infusion pump syringe clamp trays and slide-rail decorative covers.

### 2.3 3D-printing system, software, and materials

1. CAD software: AutoCAD and SOLIDWORKS 2025 are utilized for 3D modeling, with models exported in STL format.
2. Slicer software: ideaMaker (Raise3D) is employed for slicing and exporting G-code.
3. 3D printer: The Raise3D Pro3 Plus HS (Raise3D, China) is used for the printing process.
4. Filament: Industrial-grade 1.75 mm Raise3D Hyper Speed PLA is available in multiple colors, featuring a tensile strength of ≥55 MPa, a flexural strength of ≥80 MPa, and a density of approximately 1.24 g/cm^3^.
5. Metrology Tools: Digital calipers (with a resolution of 0.02 mm), micrometers, and a coordinate-measurement approach are utilized where applicable.

### 2.4 3D modeling and process optimization

#### 2.4.1 3D modeling workflow

Using OEM spare parts or intact samples as references, the components were modeled as follows:

1. Key geometric features, including length, width, height, hole diameter, wall thickness, and chamfer radius, were measured three times using calipers, and the mean values were utilized.
2. Solid modeling: Two-dimensional sketches were created in SOLIDWORKS/AutoCAD and subsequently converted into three-dimensional solids through extrusion, revolution, and Boolean operations.
3. Design adjustments: For mating interfaces, such as press-fit joints, snap-fits, and threaded holes, installation clearances were increased by 0.1 to 0.2 mm to accommodate Fused Deposition Modeling (FDM) shrinkage and layer-stepping effects (34). In this study, fillets and reinforcing ribs were incorporated at stress concentrators to enhance part strength. This aligns with reported fracture modes in polymer AM (35).
4. Export: The models were exported as STL files and imported into the slicer for orientation and support design.

#### 2.4.2 Printing parameters and iterative optimization

Based on the properties of PLA and pilot trials (36–38), the initial parameters were established as follows: (1) Layer height: 0.2 mm; (2) Infill: 30%–50% (adjusted according to expected loading); (3) Wall thickness: 1.2 mm (three perimeters); (4) Nozzle temperature: 210 °C; (5) Bed temperature: 55 °C; (6) Print speed: 50 mm/s; (7) Cooling: 80% from layer 3 onward; (8) Supports: applied only to overhangs and complex internal cavities, with priority given to removable designs.

To address unexpected warpage and dimensional inaccuracies in specific builds, printing parameters were iteratively tuned according to established polymer AM optimization protocols (10, 39–41). These strategies included reorienting prints to align the principal loading directions closer to the layer plane, adjusting infill density, and modifying perimeter counts. All adjustments and outcomes were meticulously recorded. Detailed configurations of these parameters, including native slicing software screenshots of print orientation and support settings, are provided in Supplementary Material S2.

### 2.5 Engineering performance evaluation and economic evaluation framework

Guided by the risk classification matrix outlined in Section 2.2, we designed targeted tests for F0–C0–L0 and F0–C1–L0 components. The engineering evaluation concentrated on (i) dimensional and assembly accuracy, (ii) functional usability, and (iii) in-use reliability. The objective was to verify the suitability of these components for intended low-risk use scenarios, rather than to certify materials or components for critical functions under ASTM/ISO qualification pathways.

#### 2.5.1 Dimensional accuracy (F0)

F0 denotes non-functional structural components. Core requirements are geometric consistency and host-device assembly compatibility. We measured key dimensions of printed parts via calipers and coordinate measurement techniques where appropriate. Measured values were benchmarked against OEM parts or design models.

This study defined three primary endpoints: (1) deviations in critical assembly dimensions; (2) geometric consistency of holes, snap-fit features, and contact surfaces; and (3) any interference, looseness, or necessity for post-processing during assembly. Five printed parts were randomly sampled per spare-part model. Each sample was compared to its corresponding OEM counterpart. Key dimensional error was computed as (see Equation 1):

$$\begin{array}{c} \Delta L=L_{\mathrm{PLA}}-L_{\mathrm{original}} \end{array}$$ (1)

where $L_{\mathrm{PLA}}$ denotes the measured dimension of the 3D-printed part, and $L_{\mathrm{original}}$ refers to the corresponding dimension of the OEM spare part.

Mean error was calculated for each part type. Standard deviation was also summarized by part type. For critical dimensions, the acceptance limit was set at |ΔL|≤0.5mm. Surface appearance was evaluated independently by two senior engineers. The rating considered surface flatness, visible layering, and burrs/voids. A four-tier scale was used: excellent, good, acceptable, or unacceptable.

#### 2.5.2 Functional usability (F0 with C0–C1)

For non-critical components associated with workflows or human–machine interaction, such as button caps and panel trims, functional usability tests were conducted. These tests included: (1) assessing whether the travel and tactile feedback were normal; (2) evaluating whether repeated operations led to sticking, loosening, or functional failure; and (3) for C1 parts, surface integrity was verified under expected usage conditions. Operational stability was also confirmed for C1 parts during these tests.

PLA parts were installed on the corresponding equipment, and the following metrics were recorded: (1) first-pass installation success rate, which indicates the proportion of installations completed successfully without the need for sanding or reprinting and passing functional checks; (2) adjustment rate, reflecting the proportion of parts that required minor sanding, hole reaming, or reprinting; and (3) functional rating, which comprised subjective assessments using a 5-point Likert scale, benchmarked against OEM parts, for button feel and travel, snap-fit retention, holder stability, and pad contact stability.

#### 2.5.3 In-use reliability (F0 with C0–C1; L0)

Since all included parts were classified as low-load (L0), we did not conduct extreme-condition or destructive testing. Instead, we designed targeted durability tests based on the expected frequency of clinical usage and the safety factors reported in polymer additive manufacturing studies. The simulations by part type included: (1) Button caps: subjected to 1 press per second for a cumulative total of 20,000 cycles (approximating 1 to 2 years of routine use); assessed for loosening, cracking, or degradation in travel. (2) Snap-fit/slot parts: underwent 1,000 cycles of installation and removal; evaluated for retention and permanent deformation of the snap arms (42). (3) Probe holders and cable clips: involved repeated insertion and removal along with simulated pull forces; assessed for fractures or loosening. (4) Feet, pads, and trays: experienced static loading up to 1.5 times the design working load for 24 h to verify structural integrity under normal and mild overload conditions.

Failure criteria were defined as fracture, noticeable plastic deformation, or structural damage resulting in assembly failure or functional unavailability.

#### 2.5.4 Alignment between testing strategy and risk classification

The engineering tests described above were explicitly developed under the constraint that components were limited to F0–C0/C1–L0. The primary objective was to evaluate feasibility and durability in low-risk, non-critical scenarios typical of in-hospital maintenance workflows. This approach was not intended to replace formal materials certification or the high-level safety validation required for components associated with higher risks. Components that exhibit greater functional importance, increased contact frequency, or higher mechanical loads necessitate the use of higher-grade materials, as well as more stringent requirements for biocompatibility, structural safety, and consistency verification; these considerations fall outside the scope of this study.

#### 2.5.5 Economic evaluation framework

**Figure 4:**
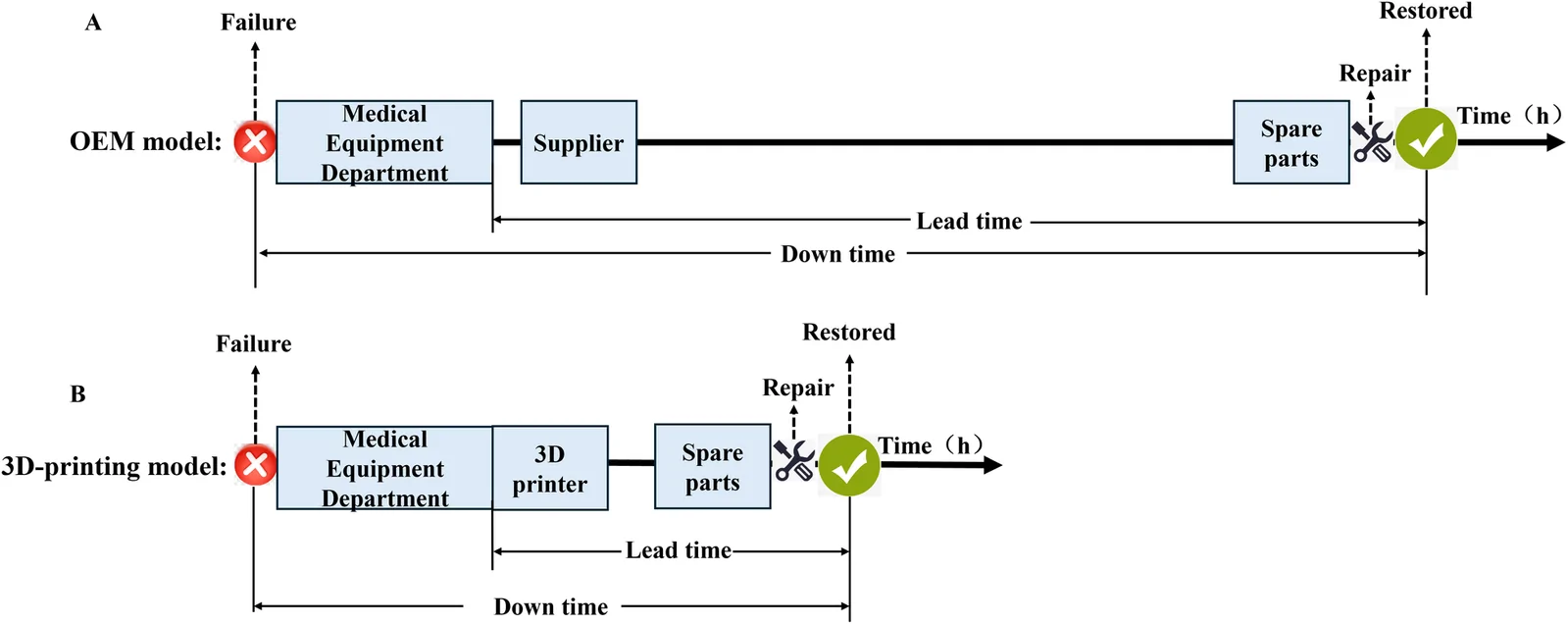
Time-sequence diagrams of the two supply modes.

Following the supply chain evaluation logic described by Chaldoupis et al. (26), this study compared an OEM procurement model with an in-house 3D printing model across four dimensions: total cost per part, spare parts lead time, equipment downtime (as shown in Figure 4), and economic benefits.

##### 2.5.5.1 Total cost per part

The total cost per part is derived from various components. The OEM part cost is calculated as the mean contract price from the hospital procurement system over the past three years.

For the 3D-printed part cost, three main components are considered: material cost $\left(C_{\mathrm{material}}\right)$, which is determined by the slicer-estimated material usage multiplied by the unit price of PLA; energy cost $\left(C_{\mathrm{energy}}\right)$, estimated based on print time and printer power consumption, with applicable electricity tariffs; and depreciation $\left(C_{\mathrm{depreciation}}\right)$, which is allocated using straight-line depreciation over three years converted to an hourly cost and then assigned based on print time.

Therefore, the total cost can be expressed as (see Equation 2):

$$\begin{array}{c} C_{\mathrm{total}}=C_{\mathrm{material}}+C_{\mathrm{energy}}+C_{\mathrm{depreciation}\;} \end{array}$$ (2)

In this study, personnel involved in 3D printing were salaried hospital employees, and as labor time was not tracked at the part level, it was not possible to allocate labor costs reliably. Consequently, labor costs were excluded from the unit-cost calculation. This approach was consistently applied across all 3D-printed parts. Thus, the reported 3D-printing unit cost reflects direct non-labor costs. The empirical proportion of these costs is approximately:

$$C_{\mathrm{material}}:C_{\mathrm{energy}}:C_{\mathrm{depreciation}\;}\approx 5:3:2$$

All costs are compared in **CNY**.

##### 2.5.5.2 Spare parts lead time

OEM model: The supply cycle was quantified as the elapsed time (in hours) from the submission of a purchase request by the Department of Medical Equipment to the arrival and installation of the spare part, rendering it ready for use (see Figure 3A). Notably, the response time from the occurrence of a failure to departmental intervention, as well as the duration from the arrival of the spare part to the completion of repairs and return to service, were both minimal and negligible in relation to the primary time scale of interest. Consequently, in subsequent analyses, equipment downtime was approximated as the spare-part lead time, i.e., $T_{\mathrm{downtime}}\approx T_{\mathrm{lead}}$.

3D-printing model: In this study, the delivery cycle for 3D-printed spare parts was defined as the total end-to-end duration experienced by the Department of Medical Equipment, encompassing the time from the receipt of the request to the completion of printing, post-processing, trial fitting/acceptance, and release for use (see Figure 3B). All spare parts included in this study had readily available three-dimensional digital models; thus, the delivery cycle did not account for the time required for initial modeling or model development.

##### 2.5.5.3 Equipment downtime and economic impact

Downtime is defined as the cumulative duration during which a device is unable to fulfill its intended clinical function and is consequently rendered out of service due to the unavailability of spare parts (see Figure 3). Utilizing hourly depreciation rates and average service revenue, this study estimated both direct and indirect economic losses associated with downtime. The hours of downtime were extracted from maintenance work orders documented in the hospital's equipment management system.

**Direct Loss (Depreciation loss):**

$$\begin{array}{c} L_{\mathrm{direct}}=T_{\mathrm{downtime}}\times R_{\mathrm{depreciation}} \end{array}$$ (3)

where $T_{\mathrm{downtime}}$ represents downtime in hours, and

$$\begin{array}{c} R_{\mathrm{depreciation}}=\frac{P_{\mathrm{equipment}}}{L_{\mathrm{year}}\times H_{\mathrm{annual}}} \end{array}$$ (4)

With $P_{\mathrm{equipment}}$ denoting the original equipment value, *L_year_* = 10 years as assumed service life, and $H_{\mathrm{annual}}=2,000\;h$ as annual operating hours.

**Indirect Loss (revenue loss):**

$$\begin{array}{c} L_{\mathrm{indirect}}=T_{\mathrm{downtime}}\times R_{\mathrm{revenue}} \end{array}$$ (5)

Where $R_{\mathrm{revenue}}$ is the mean hourly service revenue for the device category, calculated from annual financial revenue and operating hours.

**Total Loss:**

$$\begin{array}{c} L_{\mathrm{total}}=L_{\mathrm{direct}}+L_{\mathrm{indirect}} \end{array}$$ (6)

These estimates serve as a reference for order-of-magnitude calculations and do not account for more complex factors such as opportunity costs or reputational impacts; thus, the results should be interpreted conservatively.

##### 2.5.5.4 Paired before–after dataset (20 representative parts)

From the 42 eligible spare-part types, 20 representative models were selected. Selection prioritized parts with high clinical utilization and a documented shift from exclusive OEM procurement to 3D-printed replacements post-implementation. This selection encompassed various device categories, including syringe and infusion pumps, monitors, ultrasound machines, ophthalmic slit lamps, blood gas and hematology analyzers, and anesthesia machines, thereby supporting the representativeness of our findings. It should be noted that this convenience sampling strategy was strictly limited to parts with complete paired data and high utilization; consequently, it may have preferentially included parts with more favorable outcomes, potentially introducing a selection bias. For each part, we extracted the following data:

OEM model: unit purchase price, procurement lead time (in hours), and annual downtime attributable to that part (in hours).
3D-printing model: total cost per part (including materials, electricity, and depreciation), production lead time (in days), and annual downtime attributable to that part (in hours). All monetary values were expressed in CNY, and downtime was derived from the equipment management system.

#### 2.5.6 Sensitivity analysis

To evaluate the robustness of our findings beyond the base-case scenario, we conducted two sensitivity analyses (see Equations 7, 8) focusing on key cost drivers:

1. **Reduced printer utilization:** We assumed that utilization decreases to 50% of the base value, thereby increasing the allocated depreciation per unit time:
  $$\begin{array}{c} {C^{\prime }}_{\mathrm{depreciation}}=\frac{C_{\mathrm{depreciation}}}{0.5}=2\times C_{\mathrm{depreciation}} \end{array}$$ (7)
2. **Increased material cost:** We assumed that the price of PLA increases by 30%:
  $$\begin{array}{c} {C^{\prime }}_{\mathrm{material}}=1.3\times C_{\mathrm{material}} \end{array}$$ (8)

Under each scenario, we recalculated the total cost $\left(C_{\mathrm{total}}\right)$ for printed parts and compared it with OEM costs. If the 3D-printing model remained cost-advantaged in all or most scenarios, the base-case conclusions were deemed robust.

### 2.6 Statistical analysis

Analyses were conducted using SPSS version 26.0. Continuous variables are reported as mean ± standard deviation (SD), while categorical variables are expressed as counts and percentages. Primary comparisons (paired before–after, 20 parts) included total cost per part, lead time, and annual downtime, which were assessed using paired t-tests. The normality of paired differences was evaluated using the Shapiro–Wilk test; if normality was not satisfied, the Wilcoxon signed-rank test was utilized. Effect sizes for paired t-tests were calculated using Cohen's d, defined as:

$$\begin{array}{c} d=\frac{{\bar{x}}_{\mathrm{diff}}}{s_{\mathrm{diff}}} \end{array}$$ (9)

where ${\bar{x}}_{\mathrm{diff}}$ represents the mean paired difference and $s_{\mathrm{diff}}$ denotes its standard deviation.

For Wilcoxon tests, the matched-pairs rank-biserial correlation $\left(r_{\mathrm{rb}}\right)$ was reported.

Additionally, categorical variables were compared using the chi-squared(*χ*^2^) test,while Fisher's exact test was employed when expected counts were less than 5. All statistical tests were two-sided, with a significance level set at *P* < 0.05. Both *p*-values and effect sizes are reported to reflect statistical and practical significance.

## 3 Results

All 42 PLA spare-part models were successfully prototyped and optimized, achieving a first-print success rate of 85.7% (36 out of 42). For the remaining six parts, local warpage and/or undersized holes were observed. After one iteration that included adjustments to print orientation, the addition of brims, and/or minor dimensional compensation, all six parts met the acceptance requirements. The root causes of these initial failures were traced to predictable FDM process artifacts (e.g., thermal contraction-induced warpage, insufficient support for overhanging features, and uncorrected shrinkage in critical hole dimensions), all of which were resolved via standard print-parameter tuning (orientation adjustment, brim addition, and 0.1–0.2 mm dimensional compensation). A detailed tabulation of failure modes, root-cause analyses, and corrective actions is provided in Supplementary Material S1 to support clinical engineering teams adopting similar workflows.

### 3.1 Dimensional accuracy

A total of 210 critical dimension measurements were obtained (five printed samples per model × 42 models). The overall mean dimensional error was 0.18 ± 0.07 mm, with a range of 0.05 to 0.42 mm. Deviations were slightly positive (oversized), which facilitated achieving tight fits through minimal sanding when necessary. All absolute errors were within the prespecified tolerance of ±0.5 mm, indicating that the printed parts satisfied the engineering acceptance criterion. Dimensional errors categorized by spare-part type are summarized in Table 1.

**Table 1:** Critical-dimension errors for PLA FDM 3D-printed spare parts by category (*n* = 42 models; 210 measurements).

Spare-part category	No. of models	No. of samples measured	Mean error, mm (mean ± SD)	Proportion exceeding ±0.5 mm
Housing clips/fasteners	12	60	0.22 ± 0.09	0
Button caps	8	40	0.16 ± 0.05	0
Probe holders/brackets	7	35	0.19 ± 0.06	0
Cable-management clips	6	30	0.14 ± 0.04	0
Feet/pads	5	25	0.17 ± 0.08	0
Trays/guides	4	20	0.21 ± 0.09	0
Total	42	210	0.18 ± 0.07	0

### 3.2 Surface appearance

Surface appearance ratings were categorized as follows: (1) Excellent: 57.1% (24/42)—characterized by fine layer texture, flat surfaces, and the absence of visible defects; (2) Good: 35.7% (15/42)—exhibiting minor layer lines and/or localized support marks that do not impact functionality; (3) Acceptable: 7.1% (3/42)—featuring more prominent layer lines on large flat areas and small burrs, yet meeting assembly requirements after light sanding; (4) Unacceptable: 0%.

Overall, the quality of appearance was comparable to that reported in published in-hospital 3D-printing maintenance practice reports. From an engineering perspective, the observed dimensional errors fell well within typical assembly tolerances for many plastic enclosure-type components, indicating that—given appropriate modeling and process control—PLA FDM printing can adequately meet practical hospital requirements for non-critical spare parts.

### 3.3 Functional usability (assembly and user-perceived performance)

Crucially, the post-installation usability assessments were conducted by two senior clinical engineers who were independent of the 3D printing operation team (i.e., uninvolved in any stage of 3D modeling, slicing, printing, or post-processing) and remained blinded to the specific sourcing (OEM vs. in-house printed) of the installed components throughout the evaluation period. Both raters have >10 years of experience in medical equipment maintenance and are familiar with baseline OEM part performance. All components were installed and tested on their respective devices. The initial installation success rate was 92.9% (39 out of 42). Three components required minor adjustments due to tight holes or local interference; after light sanding or reprinting, all three were successfully installed. Consequently, the final installation success rate reached 100% (42 out of 42).

Utilizing a 5-point Likert scale to assess post-installation tactile feel and stability (1 = clearly inferior to OEM; 5 = superior to OEM), the overall mean score was 3.8 ± 0.6. This indicates that most users perceived the performance as either “no meaningful difference” or “slightly different but acceptable” compared to OEM parts. The distribution is presented in Table 2.

**Table 2:** Likert ratings of usability after installation (*n* = 42 parts).

Score	Response option	*n*	%	Cumulative %	Interpretation (guidance)
1	Clearly worse than OEM	0	0	0	Impairs use; rework/ replacement needed; not recommended
2	Slightly worse than OEM (usable but with obvious drawbacks)	1	2.38	2.38	Usable, but noticeable defects (e.g., difficult assembly, noise, loosening, durability concerns)
3	Comparable to OEM	8	19.05	21.43	Differences minimal or not affecting use
4	Better than OEM in some aspects	33	78.57	100	Describable advantages (easier installation, improved durability, time saving, etc.)
5	Clearly superior to OEM	0	0	100	Multiple substantial improvements; preferred option

### 3.4 In-use reliability and simulated service-life testing

It is important to clarify that the mechanical validation strategy for this study was intentionally designed to align with the risk profile of the included parts and their real-world clinical use conditions, rather than adopting generic materials-characterization protocols (e.g., ASTM tensile/impact testing). All 42 validated parts were strictly classified as L0 (low-load, non-structural) per the F-C-L matrix, with maximum expected service loads far below the tensile strength threshold of the Raise3D Hyper Speed PLA (≥55 MPa, per manufacturer datasheet). For such non-critical auxiliary components, application-specific simulated service-life tests provide greater clinical relevance than standardized material-level tests, as they capture the actual stress states, assembly constraints, and duty cycles encountered in daily hospital operations. The following tests were therefore tailored to each part category's expected usage intensity, with test durations exceeding typical annual clinical workloads to ensure adequate reliability margins.

All part categories performed well under scenario-specific simulated use tests. Eight button cap models were tested. After 20,000 press cycles, seven showed no structural damage, and one developed mild surface fatigue marks with no functional impairment. Twelve housing clip and fastener models were evaluated. After 1,000 installation–removal cycles, eleven maintained good retention; one exhibited mild permanent deformation of the clip arm but remained usable. For probe holders and cable clips (13 models), no fractures occurred during repeated insertion, removal, and pull testing, although a small number of samples showed mild plastic deformation at high-stress points. For feet, pads, and trays (9 models), after static loading at 1.5 times the design working load for 24 h, no obvious permanent deformation or cracks were detected.

Considering the devices' remaining service life and the low-load use conditions of the included parts, PLA substitutes demonstrated engineering-acceptable functional reliability over the predefined test cycles, consistent with the reported mechanical characteristics of polymer additive manufacturing components.

### 3.5 Economic evaluations

#### 3.5.1 Unit cost comparison (42 eligible models)

For the 42 eligible spare-part models, total unit costs were computed and compared between OEM procurement and in-house 3D printing (see Table 3). It is important to note that, in accordance with the Methods section, the unit cost for in-house 3D printing encompasses materials, electricity, and depreciation.

**Table 3:** Total unit costs(*n* = 42).

Cost item	OEM parts (mean ± SD), CNY	3D-printed parts (mean ± SD), CNY	Reduction, %
Total unit cost	530.92 ± 390.61	66.86 ± 57.62	87.4

#### 3.5.2 Paired before–after comparison for 20 representative parts: cost and lead time

The paired before–after dataset for 20 representative non-critical spare parts is shown in Table 4.

**Table 4:** Paired comparison of OEM procurement vs. in-house PLA FDM 3D printing for 20 representative non-critical spare parts.

Spare-part ID	Device category	Spare part	OEM unit price (CNY)	3D-printed total cost (CNY)	Annual downtime before (h)	Annual downtime after (h)
			*P* < 0.001 *rb* = 0.8766		*P* < 0.001 *rb* = 0.8769	
1	Syringe pump	Syringe clamp tray	200	5	168	12
2	Infusion pump	Housing clip A	320	5	168	15
3	ECG monitor	Button cap (menu)	200	3	240	8
4	Ultrasound	Probe cable holder	780	160	360	14
5	Ophthalmic slit lamp	Slide-rail decorative cover	450	110	168	10
6	Blood gas analyzer	Bottom foot pad	300	70	168	9
7	Hematology analyzer	Cable clip assembly	380	100	168	13
8	Anesthesia machine	Auxiliary tray	920	200	240	20
9	Syringe pump	Panel housing clip B	340	95	168	11
10	Patient monitor	Cable fixation clip	280	5	168	9
11	Ventilator	Knob cover (mode select)	520	125	240	13
12	Defibrillator monitor	Electrode-paddle holder	760	150	168	16
13	Anesthesia machine	Gas-interface protective cover	410	100	240	11
14	CT console	Keyboard keycap (function key)	360	95	240	13
15	DR workstatio	Mouse tray/small support plate	430	110	168	12
16	Syringe pump	Rail stopper block	310	85	240	9
17	Infusion pump	Power-cable clip	290	80	168	8
18	Ultrasound	Probe hook/holder	680	155	240	14
19	Ophthalmic retinoscope	Side decorative cover	390	95	168	11
20	Blood warmer	Base anti-slip foot pad	320	90	168	10
21	Mean ± SD		432 ± 195.36	91.9 ± 53.3	202.8 ± 49.46	11.9 ± 2.91

Based on the statistical analysis and Equation 9, these results indicate that, while maintaining functional equivalence for eligible low-risk parts, in-house PLA printing offers substantial advantages in both cost and timeliness, consistent with the reported supply-chain benefits of AM for spare parts. From a health-system operations perspective, shorter lead times plausibly translate into higher device availability, particularly for high-utilization imaging, monitoring, and infusion equipment. It is worth noting that the 20 representative parts analyzed here were selected based on the availability of complete paired data and high clinical utilization, which may not fully capture the variability across all 42 eligible part types.

#### 3.5.3 Downtime reduction and estimated economic benefits

For the 20 representative parts, annual equipment downtime decreased significantly from 202.8 ± 49.46 h (before implementation) to 11.9 ± 2.9 h (after implementation), reflecting a relative reduction of 94.13%.

**Figure 5:**
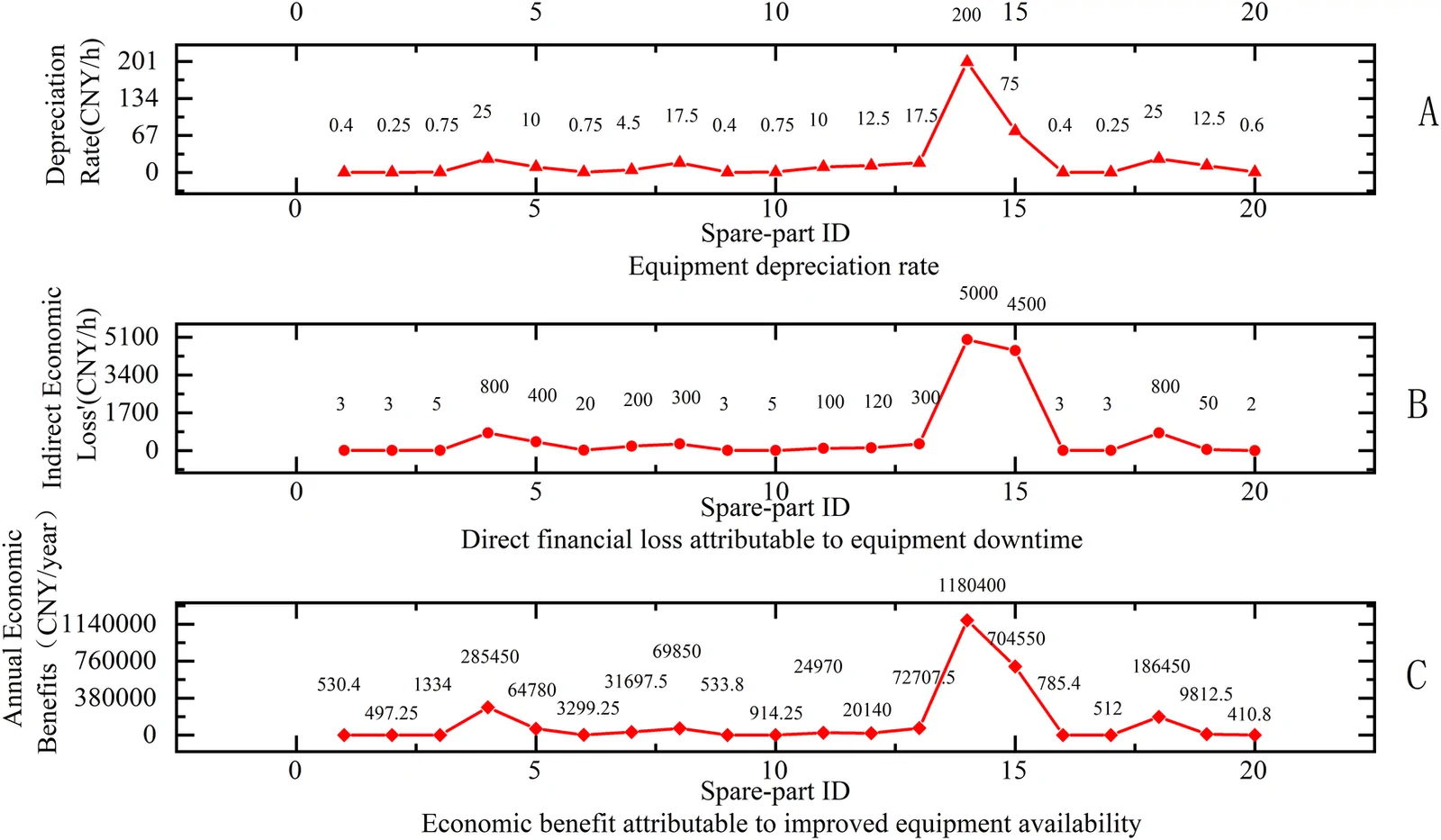
Economic impact.

Based on Equations 3–6 in Section 2.5.5, this study calculated the equipment depreciation cost, the direct economic loss attributable to equipment downtime, and the economic benefits associated with the implementation of in-house 3D printing. The detailed results are presented in Figure 5. By applying the loss-estimation approach defined in Section 2.5.5, the total annualized economic benefit attributable to the 20 parts was estimated to be CNY 2.66 million per year. Considering the estimated acquisition and operating cost of the 3D-printing program, which totals 150,000 CNY, the one-year return on investment was approximately 1:53. Given the reliance on estimated parameters (e.g., hourly revenue, asset lifespan), these economic outputs should be interpreted as order-of-magnitude estimates for strategic planning rather than precise accounting figures.

#### 3.5.4 Sensitivity analysis

To evaluate the robustness of the base-case economic conclusions beyond favorable operating conditions, we conducted a scenario-based sensitivity analysis focusing on key cost drivers of in-house 3D printing. The base-case total unit cost of CNY 66.86 represents the mean across all 42 eligible spare-part models (SD: ±57.62, Table 3), derived from the cost structure $C_{\mathrm{material}}:C_{\mathrm{energy}}:C_{\mathrm{depreciation}}\approx 5:3:2$ defined in section 2.5.5. All variable ranges were explicitly defined based on local hospital procurement records (2023–2026) and observed PLA market price volatility in China: printer utilization (plausible range: 30%–70%, reflecting variable spare-part demand and idle periods between print jobs), and PLA filament cost (plausible range: ±20%–50%, based on historical supplier pricing fluctuations). The proportional adjustments below apply uniformly to individual part costs given the linear structure of the cost model.

##### 3.5.4.1 Scenario 1: reduced printer utilization

Base-case utilization was assumed at 100% of available runtime. We modeled a decrease to 50% utilization, which doubles the allocated depreciation cost per unit. This yields (see Equation 10):

$$\begin{array}{r} \begin{array}{c} \frac{{C^{\prime }}_{\mathrm{total}}}{C_{\mathrm{total}}}=\frac{C_{\mathrm{material}}+C_{\mathrm{energy}}+2C_{\mathrm{depreciation}}}{C_{\mathrm{material}}+C_{\mathrm{energy}}+C_{\mathrm{depreciation}}}\\ =1+\frac{C_{\mathrm{depreciation}}}{C_{\mathrm{total}}}\approx 1+0.2=1.2 \end{array} \end{array}$$ (10)

This corresponds to an approximate 20% increase in unit cost (from CNY 66.86 to CNY 80.23), while the cost advantage relative to OEM parts (CNY 530.92) remains substantial at 84.9%.

##### 3.5.4.2 Scenario 2: increased PLA material price

We modeled a 30% price increase based on observed market volatility. This adjustment yields (see Equation 11):

$$\begin{array}{r} \begin{array}{c} \frac{{C^{\prime }}_{\mathrm{total}}}{C_{\mathrm{total}}}=\frac{1.3C_{\mathrm{material}}+C_{\mathrm{energy}}+C_{\mathrm{depreciation}}}{C_{\mathrm{material}}+C_{\mathrm{energy}}+C_{\mathrm{depreciation}}}\\ =1+\frac{0.3C_{\mathrm{material}}}{C_{\mathrm{total}}}\approx 1+0.15=1.15 \end{array} \end{array}$$ (11)

This corresponds to an approximate 15% increase in unit cost (from CNY 66.86 to CNY 76.89), with the cost advantage relative to OEM parts remaining at 85.5%.

##### 3.5.4.3 Scenario 3: combined worst-case scenario

To test the lower bound of economic viability, both Scenario 1 and Scenario 2 were applied simultaneously (50% utilization + 30% material cost increase), representing the most conservative operating condition (see Equation 12):

$$\begin{array}{c} {C^{\prime }}_{\mathrm{total}}=1.2C_{\mathrm{material}}+C_{\mathrm{energy}}+2C_{\mathrm{depreciation}}=1.35C_{\mathrm{total}} \end{array}$$ (12)

Even under this combined worst-case, the total unit cost increases by approximately 35% (to CNY 90.26), which remains 83.0% lower than the OEM equivalent (CNY 530.92 vs. CNY 90.26).

##### 3.5.4.4 Scenario 4: labor cost inclusion (conservative extension)

Although labor costs were excluded from the base case as personnel were salaried hospital employees, we evaluated a conservative scenario incorporating labor at CNY 30/hour for 2 h of modeling and printing labor per part (CNY 60). Even with this addition, the total unit cost reaches CNY 150.26, still 71.7% lower than OEM procurement.

These scenarios confirm that the economic benefit of in-house PLA printing is driven primarily by structural supply-chain simplification (eliminating multi-tier procurement, warehousing, and logistics costs) rather than marginal fluctuations in any single input category. The cost advantage therefore persists across all plausible operational perturbations, supporting the robustness of our economic conclusions.

## 4 Discussion

### 4.1 Engineering feasibility of in-house PLA FDM spare parts

To our knowledge, this study represents the first systematic attempt to evaluate PLA FDM 3D-printed substitutions for 42 non-critical spare parts across multiple device categories within a hospital setting. The results indicate that, with appropriate process optimization and structural design, PLA-printed parts can satisfy the substitution requirements for non-critical structural components in terms of dimensional accuracy (mean error: 0.18 ± 0.07 mm), assembly fit (first-pass success rate: 92.9%), and medium-term functional reliability. This finding aligns with the conclusions of Rouf et al. (16) regarding the performance of polymer 3D-printed parts. Importantly, by systematically testing a variety of spare-part types, our work advances prior case-based reports into a quantifiable engineering evaluation framework (19, 20, 43).

The anisotropy and thermal sensitivity of PLA FDM parts (44) necessitate explicit consideration of the alignment between loading direction and print orientation during the design phase. In this study, aligning the primary load direction parallel to the layer plane enhanced interlayer bonding strength and increased the safety margin. Future work could integrate finite element analysis and topology optimization to achieve a more refined process-structure-performance matching. Notably, the non-critical spare parts examined here primarily require dimensional stability, structural integrity, and basic durability, without the need for high-temperature operation, high mechanical loads, or complex biocompatibility requirements. Within this defined application boundary, PLA FDM offers a low-cost, fit-for-purpose manufacturing approach, underscoring. Its practical feasibility for hospital maintenance is confirmed (45).

The simulated service-life tests described above (20,000 press cycles for button caps, 1,000 installation-removal cycles for snap-fits, 1.5× static load endurance for feet/trays) represent functionally targeted mechanical validation for L0-class parts. For context, a button on a high-utilization infusion pump is pressed approximately 50–100 times per day in our clinical setting, meaning 20,000 cycles equates to 200–400 days of continuous use—effectively covering 1–2 years of real-world service life. This approach aligns with the risk-based validation principles of ISO 14971:2019, which prioritizes testing that reflects actual use conditions over generic laboratory characterization for low-risk components. While standardized ASTM/ISO mechanical testing would provide supplementary materials-level data (46), such tests were deemed unnecessary for the L0 parts in this study, whose failure modes are dominated by wear or deformation under cyclic operational loads rather than catastrophic material failure. Future studies expanding the F-C-L framework to F1/L1 components will incorporate comprehensive standardized mechanical validation as appropriate.

### 4.2 Economic impact and supply-chain resilience

This study quantified the economic advantages of 3D printing. Four dimensions were evaluated: per-part cost, supply lead time, downtime duration, and economic benefit. The results indicate that, even after accounting for equipment depreciation, the all-in unit cost of 3D-printed spare parts was 87.4% lower than that of OEM procurement (*P* < 0.001). Concurrently, supply lead time was reduced from a mean of 202.8 h to 11.9 h (*P* < 0.001, *d* = 3.68). These findings are consistent with the analysis by Chaldoupis et al (26). regarding the value of additive manufacturing in medical device supply chains. Our study contributes empirical evidence from the perspective of hospital operations and maintenance.

Sensitivity analyses further indicated that the 3D-printing model retained a cost advantage even under scenarios involving a 50% decrease in equipment utilization or a 30% increase in material costs. This suggests that the economic value of 3D printing is driven not only by lower material costs but also by the simplification of the supply chain—transforming a lengthy chain of “procurement–warehousing–distribution” into a streamlined print-to-need workflow (47, 48).

### 4.3 Implications for hospital operations and workforce capacity

Our results suggest that establishing an in-house, small-scale 3D-printing platform provides three operational and managerial benefits. First, it supports the digital transformation of spare-part management. By creating a standardized repository of 3D models, hospitals can convert portions of their physical inventory into “digital inventory,” enabling lean inventory management. This strategy is particularly suited for low-frequency, high-cost, or discontinued parts. It significantly reduces both inventory carrying costs and obsolescence losses. Second, it enhances supply-chain resilience. For discontinued device models, rarely used parts, or urgent demands, 3D printing facilitates rapid response and self-supply, thereby decreasing reliance on external suppliers. This capability proves especially valuable during public health emergencies. Third, it fosters capability building among clinical engineering staff. Operating a 3D-printing platform necessitates competencies in 3D modeling, process tuning, and basic mechanical assessment, thereby facilitating a transition from passive repair to active design and improvement (49). This aligns with the broader trend toward medical-engineering collaboration and innovation-driven development. From a public health perspective, these benefits extend beyond individual devices or departments. They support the continuity of clinical services and stable system operations, particularly during periods of high equipment workload or sudden public health events (50, 51).

### 4.4 Safety boundaries and risk governance for 3D-printed parts

The use of 3D-printed components in medical settings faces specific challenges. These include material biocompatibility, mechanical reliability, and compatibility with disinfection procedures (52). PLA's inherent limitations in thermal/hydrolytic stability were proactively embedded in the F-C-L screening framework from the outset: all eligible parts were excluded from high-temperature sterilization (53, 54), corrosive chemical exposure, or high-humidity environments per Section 2.2.3. Routine cleaning with 75% ethanol wipes—consistent with standard low-level disinfection for non-critical device surfaces and validated to minimally impact PLA mechanics in prior studies is recommended for all printed components (55). Periodic visual inspection for deformation/discoloration is advised for clinical implementation.

This study applied strict inclusion criteria (F0–C0/C1–L0) and a risk grading framework. Explicit safety boundaries were delineated. An actionable risk-control approach was provided for hospitals implementing similar initiatives (50). Beyond the engineering validation itself, a key contribution of this work lies in the F-C-L risk classification matrix, which addresses a methodological gap in the intersection of medical device maintenance and additive manufacturing. Existing risk-assessment methodologies in healthcare—such as ISO 14971:2019 for medical devices and IEC 80001-1 for IT-networked medical devices—are device-centric and focus on patient safety at the system level (56). These frameworks do not provide granular guidance for component-level substitution decisions, particularly for non-critical parts where the safety stakes differ fundamentally from those of implantable or life-support components. The F-C-L matrix fills this void by introducing a three-dimensional, component-level taxonomy (Functional, Contact, Load) that maps directly onto the operational realities of hospital workshops. Crucially, the framework transforms abstract regulatory concepts—such as “acceptable risk” and “patient contact”—into explicit, measurable thresholds (e.g., F0, L0, C ≤ C1) that can be applied by technicians without specialized regulatory expertise. This represents a departure from conventional risk scoring systems (e.g., FMEA's RPN calculations) (57), which are optimized for design-phase decision-making rather than rapid, *in-situ* maintenance triage. By providing a reproducible, binary eligibility rule set, the F-C-L framework enables hospitals to systematically explore 3D printing as a supply-chain alternative while maintaining clear safety boundaries—a balance that existing general-purpose risk tools do not achieve.

To support safe scale-up, the study recommend the following governance mechanisms: Hospitals should establish a risk-graded spare-part catalogue that clearly defines high-risk component categories where 3D-printed substitution is prohibited (e.g., components related to electrical insulation, radiation shielding, or high load-bearing functions). For moderate-risk components, a more stringent verification and validation pathway should be required. Additionally, standardized technical specifications should be developed for the end-to-end workflow—data acquisition, modeling, printing, post-processing, assembly, and traceability—incorporating harmonized documentation, key parameters, and inspection items informed by prior maintenance-related 3D printing practices. Cleaning and disinfection methods should also be standardized: PLA is unsuitable for high-temperature steam sterilization or strongly corrosive chemical disinfectants; thus, recommended wipe-based and low-temperature methods should be explicitly stated (58).

Future studies should systematically quantify the effects of disinfection on PLA mechanical performance (59). Finally, ethics and intellectual property governance should be strengthened (60). Although most parts in this study were discontinued by manufacturers or are generic structural components, model provenance and IP considerations also apply to parts within active supply cycles to prevent inappropriate replication (61).

### 4.5 Limitations and future directions

Several limitations must be acknowledged. First, this study was conducted at a single center; although the framework is broadly applicable, the data reflect local conditions. Nonetheless, the types of spare parts included (e.g., housings, clips, brackets) and their usage contexts are common across comparable hospitals, suggesting that the risk grading concept and evaluation framework can be transferred. Second, only PLA FDM was evaluated; other materials and printing processes were not assessed. Third, long-term reliability data throughout the entire life cycle remain limited. Fourth, the economic estimation included simplifying assumptions, and indirect economic losses were estimated using average parameters, which may potentially understate real-world impacts. Fifth, the selection of 20 representative parts for detailed economic analysis was not randomized but based on the availability of complete paired data. This introduces a potential selection bias favoring parts with successful implementation histories, which may overestimate the real-world cost-saving potential. Sixth, the before-after design lacks a concurrent control group. Consequently, while the 94.1% reduction in downtime correlates strongly with the intervention, we cannot fully exclude contributions from secular trends, such as broader supply-chain recoveries post-COVID-19 or unrelated improvements in hospital logistics and procurement contracts during the 2022–2026 study window.

Future research could prioritize: (1) systematic studies on material–process–performance relationships assessing various materials under different disinfection methods; (2) extending the analysis to moderate-risk spare parts to clarify the boundaries for F1 or L1 level components; (3) integrating 3D printing with digital twins and predictive maintenance through equipment health monitoring systems; and (4) developing regional sharing models within the Chinese tertiary hospital ecosystem, such as establishing cross-hospital digital spare-part repositories and mechanisms for experience sharing.

## 5 Conclusion

This study presents three primary contributions. Theoretically, the study proposed the F–C–L risk grading matrix for medical equipment spare parts. This framework establishes a methodological basis for the safe adoption of 3D printing. Practically, systematic engineering tests and economic analyses were conducted. We validated the feasibility and value of PLA FDM 3D printing for non-critical spare parts in hospital settings. From a managerial perspective, additive manufacturing was integrated into hospital operations and maintenance. This facilitates a transition from external supply-chain dependence to in-house manufacturing capabilities.

Overall, our findings confirm that 3D printing empowers hospitals to enhance equipment support and optimize supply-chain structures, provided that clear safety boundaries and robust quality controls are maintained. As technology matures and standards evolve, broader adoption of this model in medical contexts is anticipated. From a health-system viewpoint, minimizing unplanned equipment downtime improves continuity of care and resource utilization. The significance of this approach extends beyond engineering cost savings to bolster the overall operational resilience of hospitals.

## Data Availability

The raw data supporting the conclusions of this article will be made available by the authors, without undue reservation.
