# Supplementary material for: A risk-stratified framework for in-house PLA 3D printing of non-critical medical device spare parts: a cost-effectiveness analysis
Source: Front Med (Lausanne). 2026 Aug 27;13:1911165. doi: 10.3389/fmed.2026.1911165 (PMC13561092; doi:10.3389/fmed.2026.1911165)
Supplement: Supplementary file 1 [file Datasheet1.pdf]

Purpose: This supplementary material provides a detailed tabulation of the six initially unsuccessful prints referenced in Section 3 of the main manuscript. The analysis is intended to serve as a practical reference for clinical engineering teams implementing in-house 3D printing programs for non-critical medical device spare parts, and does not alter the core conclusions of the primary study.

S1.1 Overview of Initial Print Failures

Among the 42 spare-part models evaluated, six models (14.3%) did not meet the predefined dimensional acceptance criterion (±0.5 mm) on the first printing attempt, resulting in an initial success rate of 85.7% (or 92.9% if counted by individual instances). All six cases were resolved after one iteration of process adjustment, achieving a final installation success rate of 100%. The root causes were systematically traced to common FDM process artifacts rather than fundamental material unsuitability of PLA.

S1.2 Failure Mode, Root-Cause, and Corrective Action Table

Case ID	Part Type / Device	Failure Mode	Root Cause (Engineering Observation & Mechanism)	Corrective Action	Final Dimensional Error
S1-01	Housing clip / Monitor	Corner warpage and local lifting	**Thermal contraction-induced warpage.** Large flat base cooled faster at edges than center, generating differential shrinkage stress that exceeded bed adhesion. This is a typical FDM defect caused by non-uniform thermal contraction and internal residual stress.	Added 5-mm brim; increased bed temperature from 55°C to 60°C; reduced first-layer speed to 30 mm/s.	0.31 mm (passed)
S1-02	Button cap / Infusion pump	Undersized mounting hole (−0.4 mm)	**Uncompensated PLA thermal shrinkage.** Circular apertures are prone to dimensional underestimation because extruded filament bulges inward upon cooling; common compensation range is −0.1 to −0.5 mm.	Applied 0.2 mm hole-expansion compensation in CAD; verified with caliper measurement.	0.12 mm (passed)
S1-03	Probe holder / Ultrasound	Support scar interfering with snap-fit	**Suboptimal support placement.** Dense linear supports at critical mating surfaces left residual marks and local dimensional deviation after removal. Improper support increases post-processing variability and affects assembly fit.	Reoriented print to minimize overhangs; switched to tree supports; placed supports away from mating surfaces.	0.22 mm (passed)

S1-04	Auxiliary tray / Anesthesia machine	Edge lifting on large flat area	**Bed adhesion failure with thin-wall geometry.** Long peripheral perimeters accumulated cumulative shrinkage stress; first layer partially detached during later stages. Large flat geometries concentrate stress at corners and are sensitive to draft and cooling rate.	Enabled draft shield in slicer; increased bed temperature to 60°C; added brim ears at sharp corners.	0.28 mm (passed)
S1-05	Rail stopper / Syringe pump	Dimensional mismatch at mating surface	**Measurement error during reverse modeling.** Original caliper measurement of OEM part underestimated a 0.3-mm chamfer radius, leading to CAD-model deviation. Reverse engineering of small features is a common source of dimensional error in spare-part printing.	Remeasured OEM part with digital caliper; corrected CAD model.	0.15 mm (passed)
S1-06	Foot pad / Blood warmer	Brittle layer separation at stress concentration	**Insufficient interlayer bonding.** Localized overcooling and excessive part-cooling fan speed reduced interlayer fusion at a thin-transition zone, causing micro-delamination under handling stress. Layer separation is linked to low nozzle temperature, excessive cooling, and high thermal gradient.	Increased nozzle temperature from 210°C to 215°C; reduced cooling fan to 60% after layer 3; added one extra perimeter.	0.19 mm (passed)

S1.3 Summary of Process Lessons for Clinical Engineering Teams

The failure patterns observed in this study are consistent with commonly reported FDM process artifacts and can be mitigated through standard parameter tuning rather than material substitution:

1.Thermal management — PLA exhibits lower shrinkage than ABS or nylon but remains susceptible to differential cooling on large flat parts. Bed temperature stabilization (55–65°C), draft avoidance, and reduced first-layer cooling effectively suppress warpage.

2.Dimensional compensation — Mounting holes and snap-fit features should receive 0.1–0.2 mm compensatory allowance during CAD modeling, as aperture shrinkage is a recurrent FDM accuracy issue.

3.Orientation and support optimization — Critical mating surfaces should be oriented away from support contact; tree supports are preferred over dense linear supports for internal geometries.

4.Reverse-modeling verification — Physical remeasurement of OEM parts is recommended when CAD models are reconstructed from caliper data, particularly for chamfers, fillets, and hidden features.

5.Iterative correction is intrinsic to in-hospital printing — All failures in this study were resolved within one iteration, confirming that in-house 3D printing of L0-class non-critical spare parts is tolerant to first-attempt errors and amenable to rapid engineering correction.

S1.4 Statement on Core Study Focus

The failure analysis and corrective actions presented in this supplementary material represent context-specific operational experience derived from our single-center implementation using the Raise3D Pro3 Plus HS printer and the 42 non-critical spare-part geometries evaluated in this study. While generalizable process-tuning principles (e.g., dimensional compensation for shrinkage, thermal management for warpage) are highlighted, the magnitude of parameter adjustments may vary across different FDM printer models, filament batches, and clinical engineering workshop environments. Users adopting similar workflows are advised to perform site-specific validation for their own equipment and spare-part types, rather than directly applying these values as universal standards. The primary conclusions of the main manuscript—namely, the validity of the F-C-L risk-classification framework and the economic viability of in-house printing—remain unchanged by these context-specific nuances.

The detailed failure analysis above is provided to enhance reproducibility and practical utility for clinical engineers. It does not imply material-level limitations of PLA that would require extended polymer-science investigation. The primary conclusions of the main manuscript—namely, the validity of the F-C-L risk-classification framework, the dimensional and functional suitability of PLA-printed L0 spare parts, and the economic advantage of in-house printing—remain unchanged.
