# Supplementary material for: A risk-stratified framework for in-house PLA 3D printing of non-critical medical device spare parts: a cost-effectiveness analysis
Source: Front Med (Lausanne). 2026 Aug 27;13:1911165. doi: 10.3389/fmed.2026.1911165 (PMC13561092; doi:10.3389/fmed.2026.1911165)
Supplement: Supplementary file 2 [file Datasheet2.pdf]

Purpose: This supplementary material provides visual documentation of the benchmark Fused Deposition Modeling (FDM) process parameters used for all 42 non-critical spare-part models evaluated in the main manuscript, in direct response to the reviewer's request for transparency regarding print orientation, infill density, layer height, and other key settings.

Context note: The parameters presented here are site-specific operational configurations for the Raise3D Pro3 Plus HS printer and Raise3D Hyper Speed PLA filament used in our tertiary hospital workshop. While the general parameter ranges (e.g., 0.2mm layer height, 30–50% infill density) are consistent with best practices for PLA FDM printing, exact values may require adjustment for different printer models, filament batches, or part geometries. Users should perform local validation prior to implementation.

S2.1 Baseline Parameter Configuration

Figure S2-1 shows the native parameter interface of the ideaMaker slicing software (Raise3D), displaying the benchmark settings applied to the majority of spare-part models in this study. Key parameters highlighted include:

Layer height: 0.2 mm (balance of surface quality and print speed)

Infill density: 30–50% (adjusted based on expected load, e.g., 30% for cable clips, 50% for small trays)

Wall thickness: 1.2 mm (3 perimeters, enhancing structural integrity for snap-fit features)

Nozzle temperature: 210°C; Bed temperature: 55°C (optimized for Raise3D Hyper Speed PLA)

Print speed: 50 mm/s (reduced to 30–35 mm/s for first 10 layers of large flat parts to mitigate warpage)

Cooling: 80% activation from layer 3 onward (minimizing thermal distortion while maintaining interlayer bonding)

Supports: Automatically generated for overhangs >45°, with tree supports prioritized to reduce post-processing residue on mating surfaces.

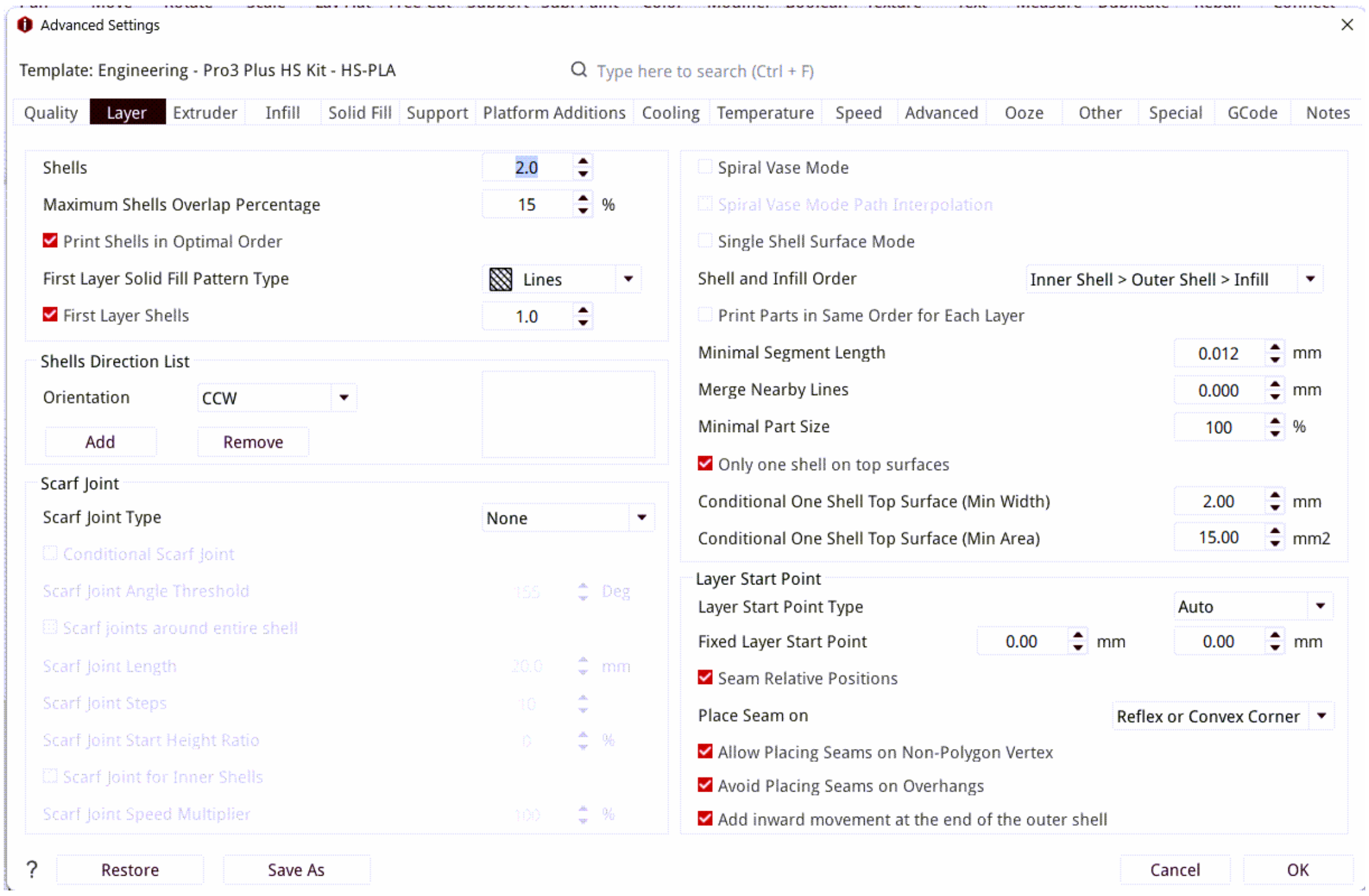

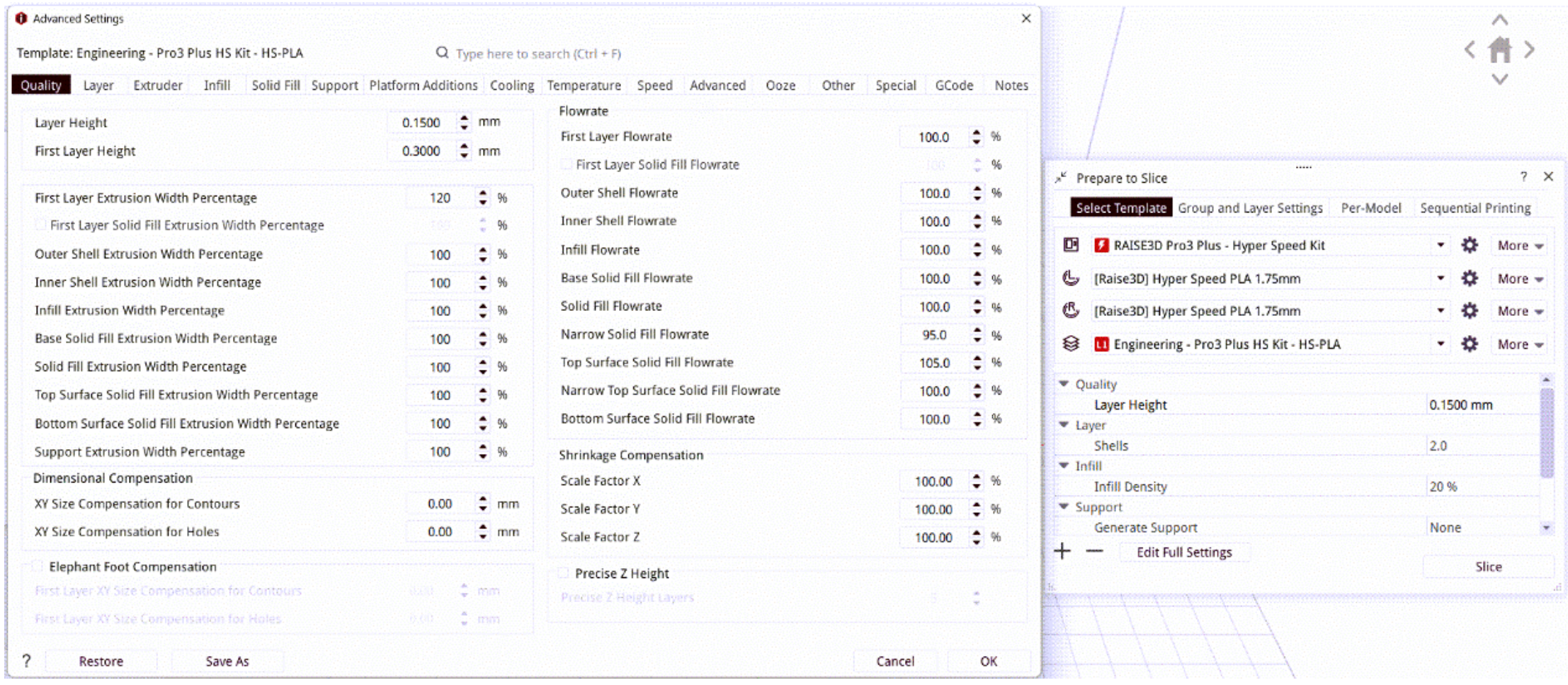
Advanced Settings
Template: Engineering - Pro3 Plus HS Kit - HS-PLA
Type here to search (Ctrl + F)
Quality Layer Extruder Infill Solid Fill Support Platform Additions Cooling Temperature Speed Advanced Ooze Other Special GCode Notes
Layer Height 0.1500 mm
First Layer Height 0.3000 mm
First Layer Extrusion Width Percentage 120 %
First Layer Solid Fill Extrusion Width Percentage
Outer Shell Extrusion Width Percentage 100 %
Inner Shell Extrusion Width Percentage 100 %
Infill Extrusion Width Percentage 100 %
Base Solid Fill Extrusion Width Percentage 100 %
Solid Fill Extrusion Width Percentage 100 %
Top Surface Solid Fill Extrusion Width Percentage 100 %
Bottom Surface Solid Fill Extrusion Width Percentage 100 %
Support Extrusion Width Percentage 100 %
Dimensional Compensation
XY Size Compensation for Contours 0.00 mm
XY Size Compensation for Holes 0.00 mm
Elephant Foot Compensation
First Layer XY Size Compensation for Contours
First Layer XY Size Compensation for Holes
Flowrate
First Layer Flowrate 100.0 %
First Layer Solid Fill Flowrate
Outer Shell Flowrate 100.0 %
Inner Shell Flowrate 100.0 %
Infill Flowrate 100.0 %
Base Solid Fill Flowrate 100.0 %
Solid Fill Flowrate 100.0 %
Narrow Solid Fill Flowrate 95.0 %
Top Surface Solid Fill Flowrate 105.0 %
Narrow Top Surface Solid Fill Flowrate 100.0 %
Bottom Surface Solid Fill Flowrate 100.0 %
Shrinkage Compensation
Scale Factor X 100.00 %
Scale Factor Y 100.00 %
Scale Factor Z 100.00 %
Precise Z Height
Precise Z Height Layers
Restore Save As Cancel OK
Prepare to Slice
Select Template Group and Layer Settings Per-Model Sequential Printing
RAISE3D Pro3 Plus - Hyper Speed Kit More
[Raise3D] Hyper Speed PLA 1.75mm More
[Raise3D] Hyper Speed PLA 1.75mm More
Engineering - Pro3 Plus HS Kit - HS-PLA More
Quality
Layer Height 0.1500 mm
Layer
Shells 2.0
Infill
Infill Density 20 %
Support
Generate Support None
Edit Full Settings
Slice

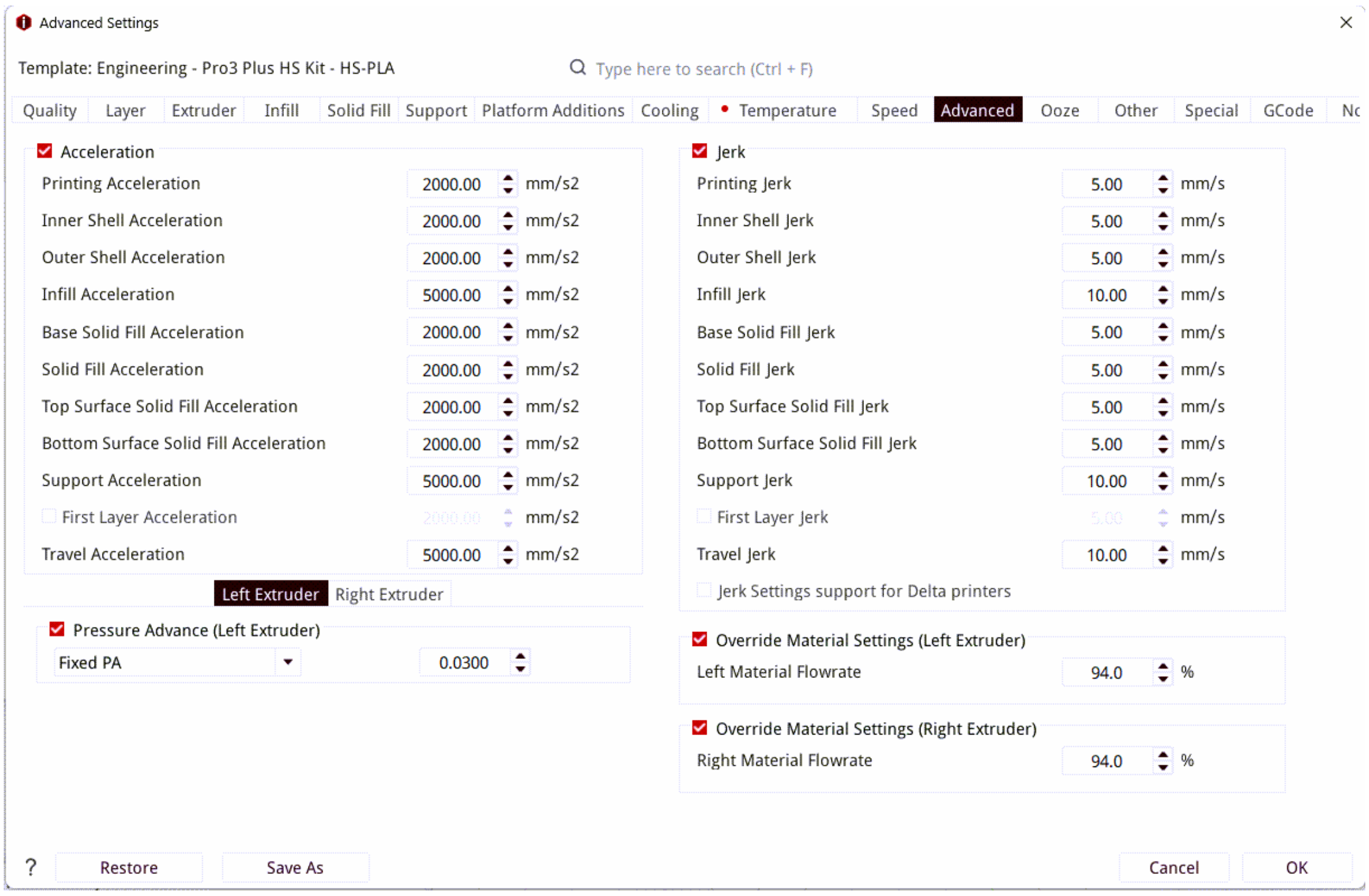
Advanced Settings
Template: Engineering - Pro3 Plus HS Kit - HS-PLA
Type here to search (Ctrl + F)
Quality Layer Extruder Infill Solid Fill Support Platform Additions Cooling Temperature Speed Advanced Ooze Other Special GCode Nc
Acceleration
Printing Acceleration 2000.00 mm/s2
Inner Shell Acceleration 2000.00 mm/s2
Outer Shell Acceleration 2000.00 mm/s2
Infill Acceleration 5000.00 mm/s2
Base Solid Fill Acceleration 2000.00 mm/s2
Solid Fill Acceleration 2000.00 mm/s2
Top Surface Solid Fill Acceleration 2000.00 mm/s2
Bottom Surface Solid Fill Acceleration 2000.00 mm/s2
Support Acceleration 5000.00 mm/s2
First Layer Acceleration 2000.00 mm/s2
Travel Acceleration 5000.00 mm/s2
Left Extruder Right Extruder
Pressure Advance (Left Extruder)
Fixed PA 0.0300
Jerk
Printing Jerk 5.00 mm/s
Inner Shell Jerk 5.00 mm/s
Outer Shell Jerk 5.00 mm/s
Infill Jerk 10.00 mm/s
Base Solid Fill Jerk 5.00 mm/s
Solid Fill Jerk 5.00 mm/s
Top Surface Solid Fill Jerk 5.00 mm/s
Bottom Surface Solid Fill Jerk 5.00 mm/s
Support Jerk 10.00 mm/s
First Layer Jerk 5.00 mm/s
Travel Jerk 10.00 mm/s
Jerk Settings support for Delta printers
Override Material Settings (Left Extruder)
Left Material Flowrate 94.0 %
Override Material Settings (Right Extruder)
Right Material Flowrate 94.0 %
?
Restore
Save As
Cancel
OK

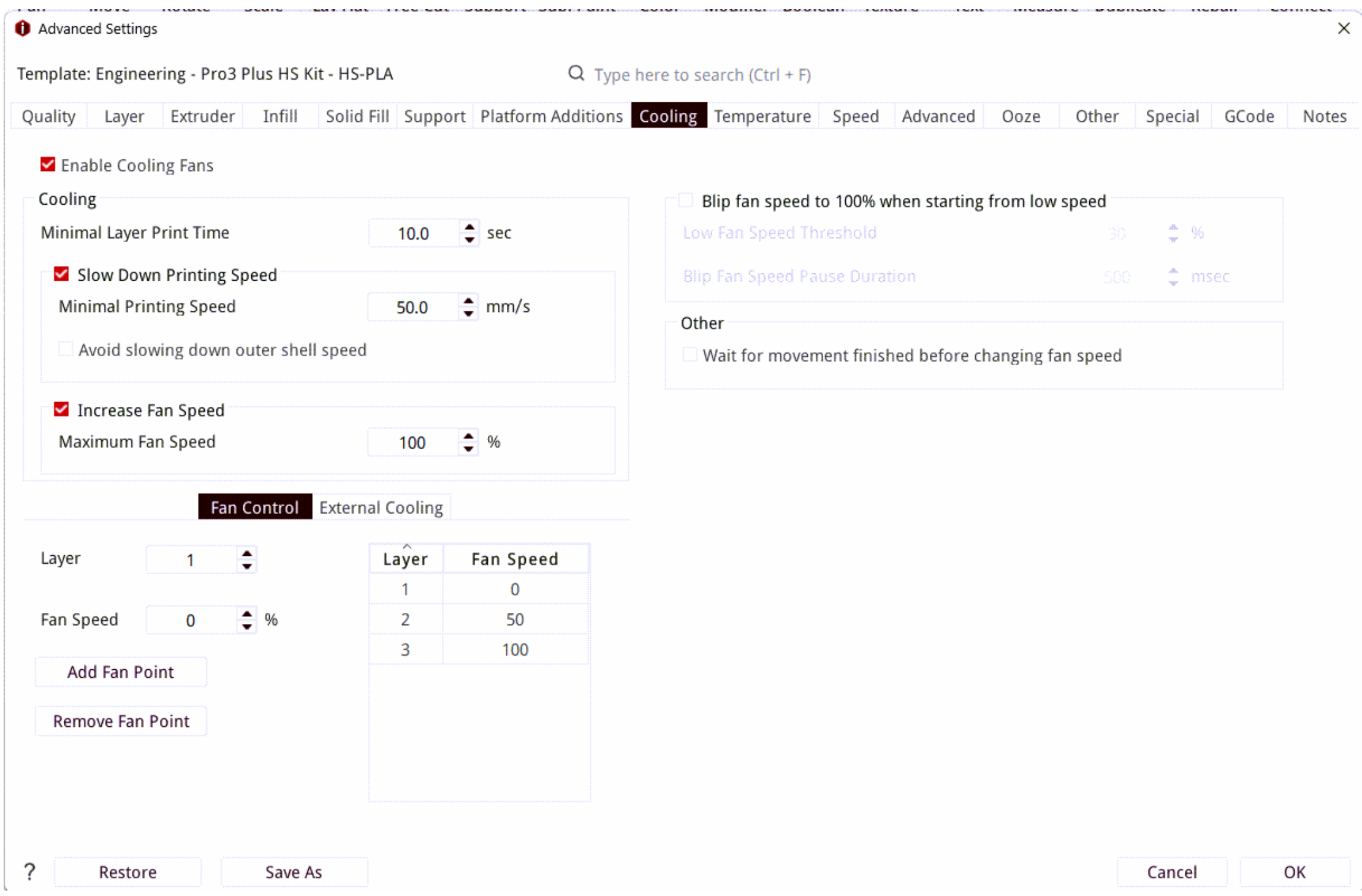
Advanced Settings
Template: Engineering - Pro3 Plus HS Kit - HS-PLA
Type here to search (Ctrl + F)
Quality
Layer
Extruder
Infill
Solid Fill
Support
Platform Additions
Cooling
Temperature
Speed
Advanced
Ooze
Other
Special
GCode
Notes
Enable Cooling Fans
Cooling
Minimal Layer Print Time 10.0 sec
Slow Down Printing Speed
Minimal Printing Speed 50.0 mm/s
Avoid slowing down outer shell speed
Increase Fan Speed
Maximum Fan Speed 100 %
Blip fan speed to 100% when starting from low speed
Low Fan Speed Threshold 30 %
Blip Fan Speed Pause Duration 500 msec
Other
Wait for movement finished before changing fan speed
Fan Control
External Cooling
Layer 1
Fan Speed 0 %
Add Fan Point
Remove Fan Point
Layer | Fan Speed
1 | 0
2 | 50
3 | 100
?
Restore
Save As
Cancel
OK

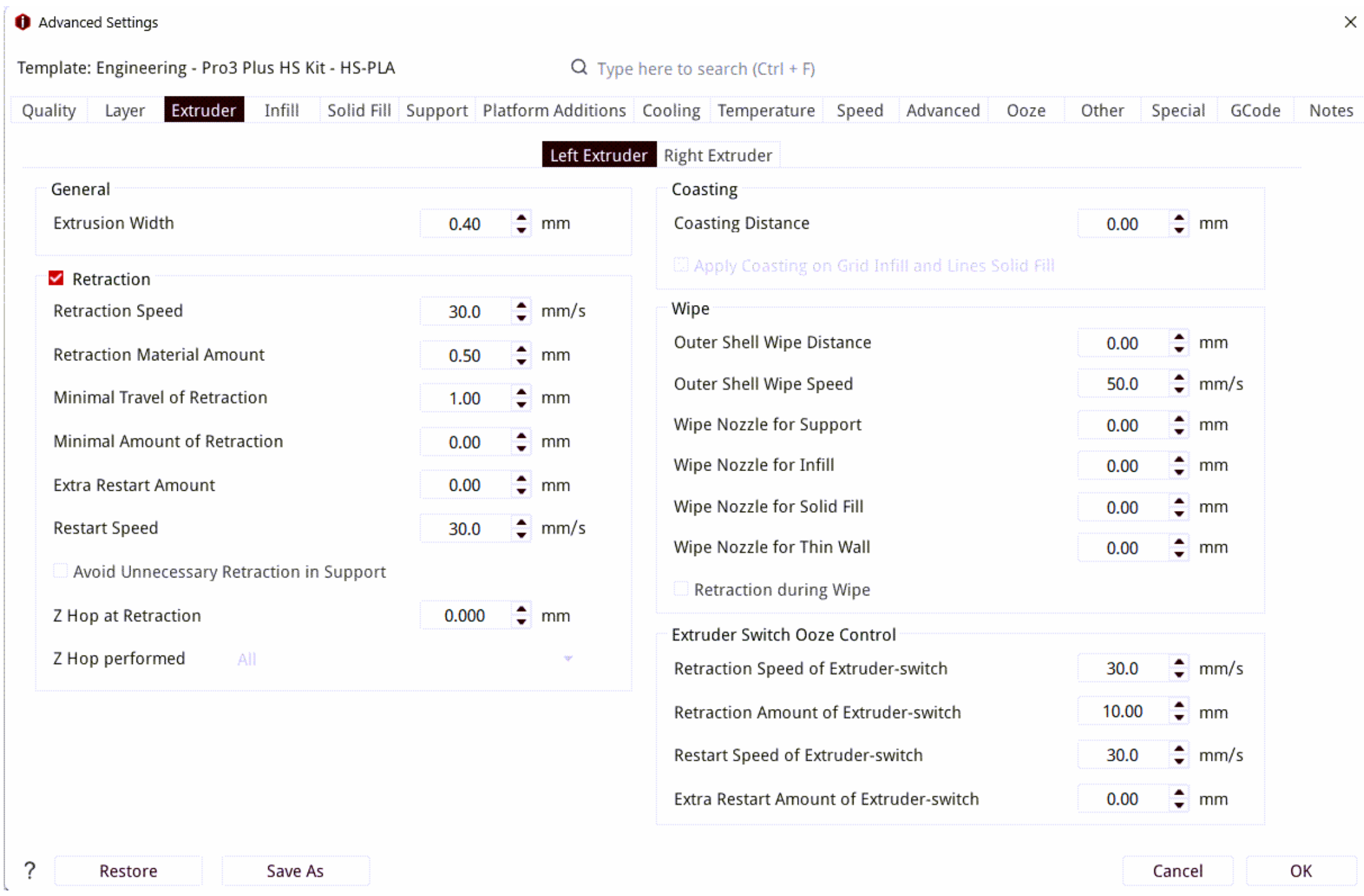
Advanced Settings
Template: Engineering - Pro3 Plus HS Kit - HS-PLA
Type here to search (Ctrl + F)
Quality
Layer
Extruder
Infill
Solid Fill
Support
Platform Additions
Cooling
Temperature
Speed
Advanced
Ooze
Other
Special
GCode
Notes
Left Extruder
Right Extruder
General
Extrusion Width 0.40 mm
Retraction
Retraction Speed 30.0 mm/s
Retraction Material Amount 0.50 mm
Minimal Travel of Retraction 1.00 mm
Minimal Amount of Retraction 0.00 mm
Extra Restart Amount 0.00 mm
Restart Speed 30.0 mm/s
Avoid Unnecessary Retraction in Support
Z Hop at Retraction 0.000 mm
Z Hop performed All
Coasting
Coasting Distance 0.00 mm
Apply Coasting on Grid Infill and Lines Solid Fill
Wipe
Outer Shell Wipe Distance 0.00 mm
Outer Shell Wipe Speed 50.0 mm/s
Wipe Nozzle for Support 0.00 mm
Wipe Nozzle for Infill 0.00 mm
Wipe Nozzle for Solid Fill 0.00 mm
Wipe Nozzle for Thin Wall 0.00 mm
Retraction during Wipe
Extruder Switch Ooze Control
Retraction Speed of Extruder-switch 30.0 mm/s
Retraction Amount of Extruder-switch 10.00 mm
Restart Speed of Extruder-switch 30.0 mm/s
Extra Restart Amount of Extruder-switch 0.00 mm
?
Restore
Save As
Cancel
OK

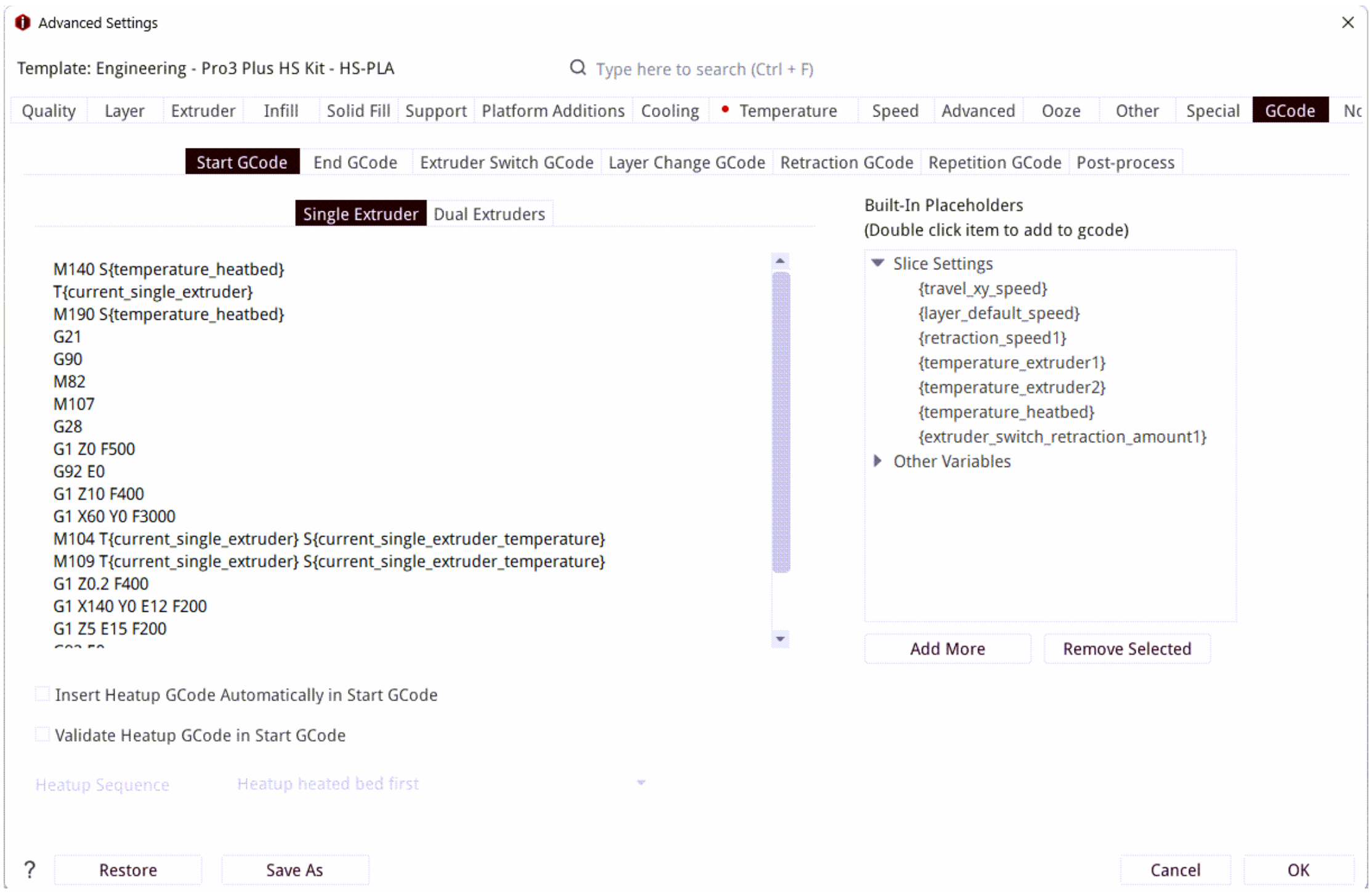
Advanced Settings
Template: Engineering - Pro3 Plus HS Kit - HS-PLA
Type here to search (Ctrl + F)
Quality Layer Extruder Infill Solid Fill Support Platform Additions Cooling Temperature Speed Advanced Ooze Other Special GCode
Start GCode End GCode Extruder Switch GCode Layer Change GCode Retraction GCode Repetition GCode Post-process
Single Extruder Dual Extruders
M140 S{temperature_heatbed}
T{current_single_extruder}
M190 S{temperature_heatbed}
G21
G90
M82
M107
G28
G1 Z0 F500
G92 E0
G1 Z10 F400
G1 X60 Y0 F3000
M104 T{current_single_extruder} S{current_single_extruder_temperature}
M109 T{current_single_extruder} S{current_single_extruder_temperature}
G1 Z0.2 F400
G1 X140 Y0 E12 F200
G1 Z5 E15 F200
Built-In Placeholders
(Double click item to add to gcode)
Slice Settings
{travel_xy_speed}
{layer_default_speed}
{retraction_speed1}
{temperature_extruder1}
{temperature_extruder2}
{temperature_heatbed}
{extruder_switch_retraction_amount1}
Other Variables
Add More
Remove Selected
Insert Heatup GCode Automatically in Start GCode
Validate Heatup GCode in Start GCode
Heatup Sequence
Heatup heated bed first
?
Restore
Save As
Cancel
OK

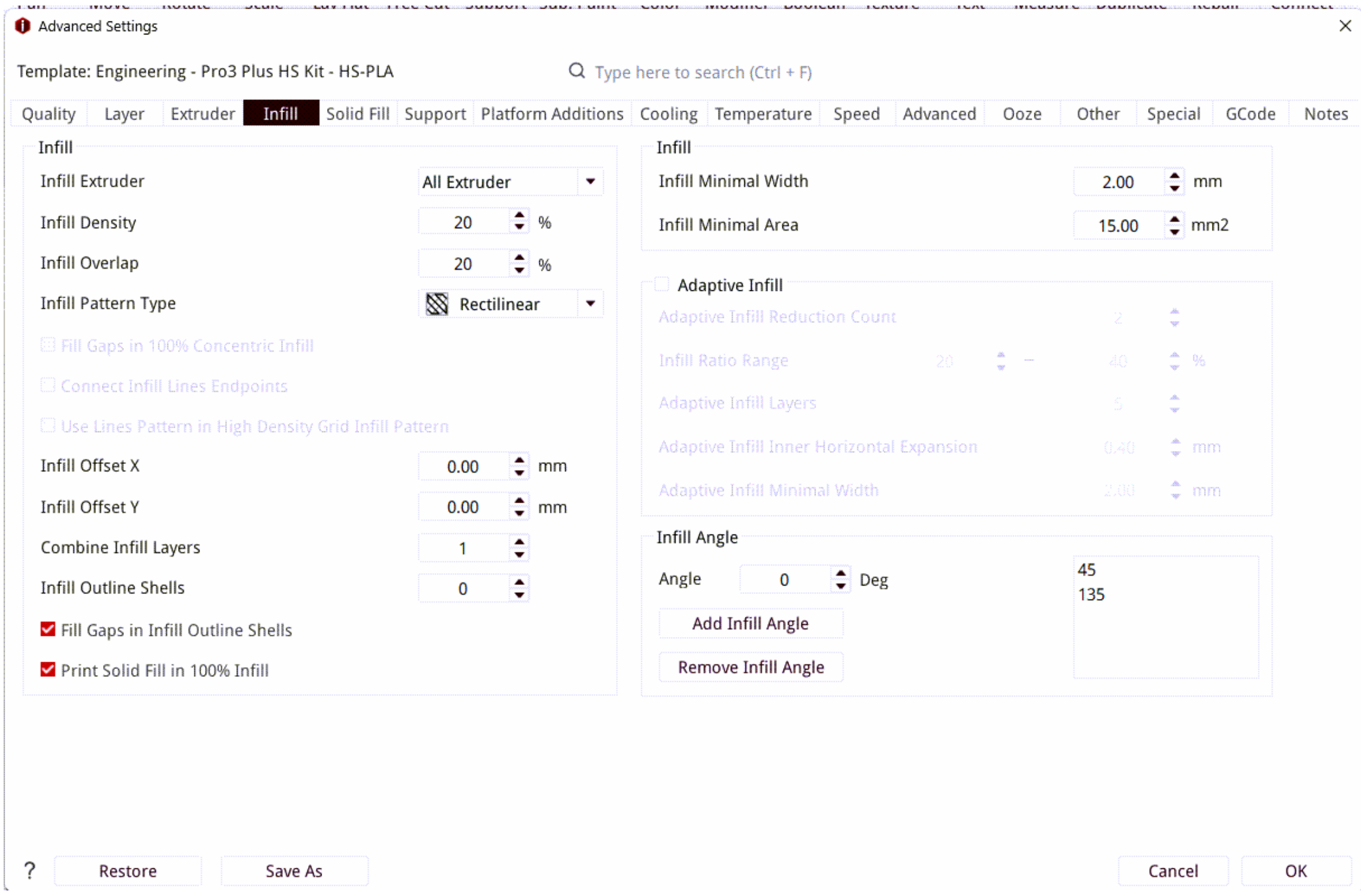
Advanced Settings
Template: Engineering - Pro3 Plus HS Kit - HS-PLA
Type here to search (Ctrl + F)
Quality Layer Extruder Infill Solid Fill Support Platform Additions Cooling Temperature Speed Advanced Ooze Other Special GCode Notes
Infill
Infill Extruder All Extruder
Infill Density 20 %
Infill Overlap 20 %
Infill Pattern Type Rectilinear
Fill Gaps in 100% Concentric Infill
Connect Infill Lines Endpoints
Use Lines Pattern in High Density Grid Infill Pattern
Infill Offset X 0.00 mm
Infill Offset Y 0.00 mm
Combine Infill Layers 1
Infill Outline Shells 0
Fill Gaps in Infill Outline Shells
Print Solid Fill in 100% Infill
Infill
Infill Minimal Width 2.00 mm
Infill Minimal Area 15.00 mm2
Adaptive Infill
Adaptive Infill Reduction Count
Infill Ratio Range
Adaptive Infill Layers
Adaptive Infill Inner Horizontal Expansion
Adaptive Infill Minimal Width
Infill Angle
Angle 0 Deg
45
135
Add Infill Angle
Remove Infill Angle
?
Restore
Save As
Cancel
OK

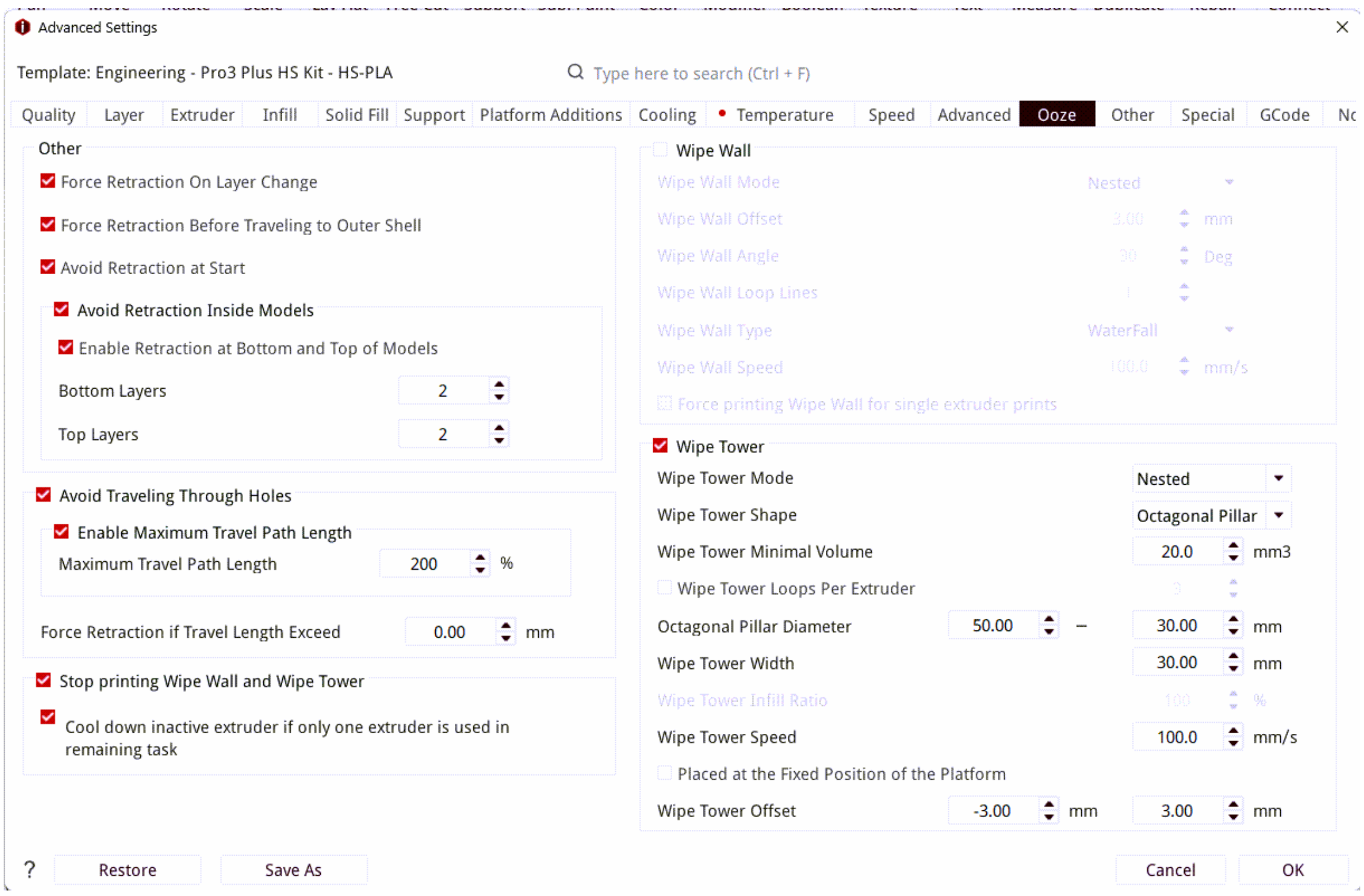
Advanced Settings
Template: Engineering - Pro3 Plus HS Kit - HS-PLA
Type here to search (Ctrl + F)
Quality
Layer
Extruder
Infill
Solid Fill
Support
Platform Additions
Cooling
Temperature
Speed
Advanced
Ooze
Other
Special
GCode
Other
Force Retraction On Layer Change
Force Retraction Before Traveling to Outer Shell
Avoid Retraction at Start
Avoid Retraction Inside Models
Enable Retraction at Bottom and Top of Models
Bottom Layers 2
Top Layers 2
Avoid Traveling Through Holes
Enable Maximum Travel Path Length
Maximum Travel Path Length 200 %
Force Retraction if Travel Length Exceed 0.00 mm
Stop printing Wipe Wall and Wipe Tower
Cool down inactive extruder if only one extruder is used in remaining task
Wipe Wall
Wipe Wall Mode Nested
Wipe Wall Offset
Wipe Wall Angle
Wipe Wall Loop Lines
Wipe Wall Type WaterFall
Wipe Wall Speed
Force printing Wipe Wall for single extruder prints
Wipe Tower
Wipe Tower Mode Nested
Wipe Tower Shape Octagonal Pillar
Wipe Tower Minimal Volume 20.0 mm3
Wipe Tower Loops Per Extruder
Octagonal Pillar Diameter 50.00 – 30.00 mm
Wipe Tower Width 30.00 mm
Wipe Tower Infill Ratio
Wipe Tower Speed 100.0 mm/s
Placed at the Fixed Position of the Platform
Wipe Tower Offset -3.00 mm 3.00 mm
?
Restore
Save As
Cancel
OK

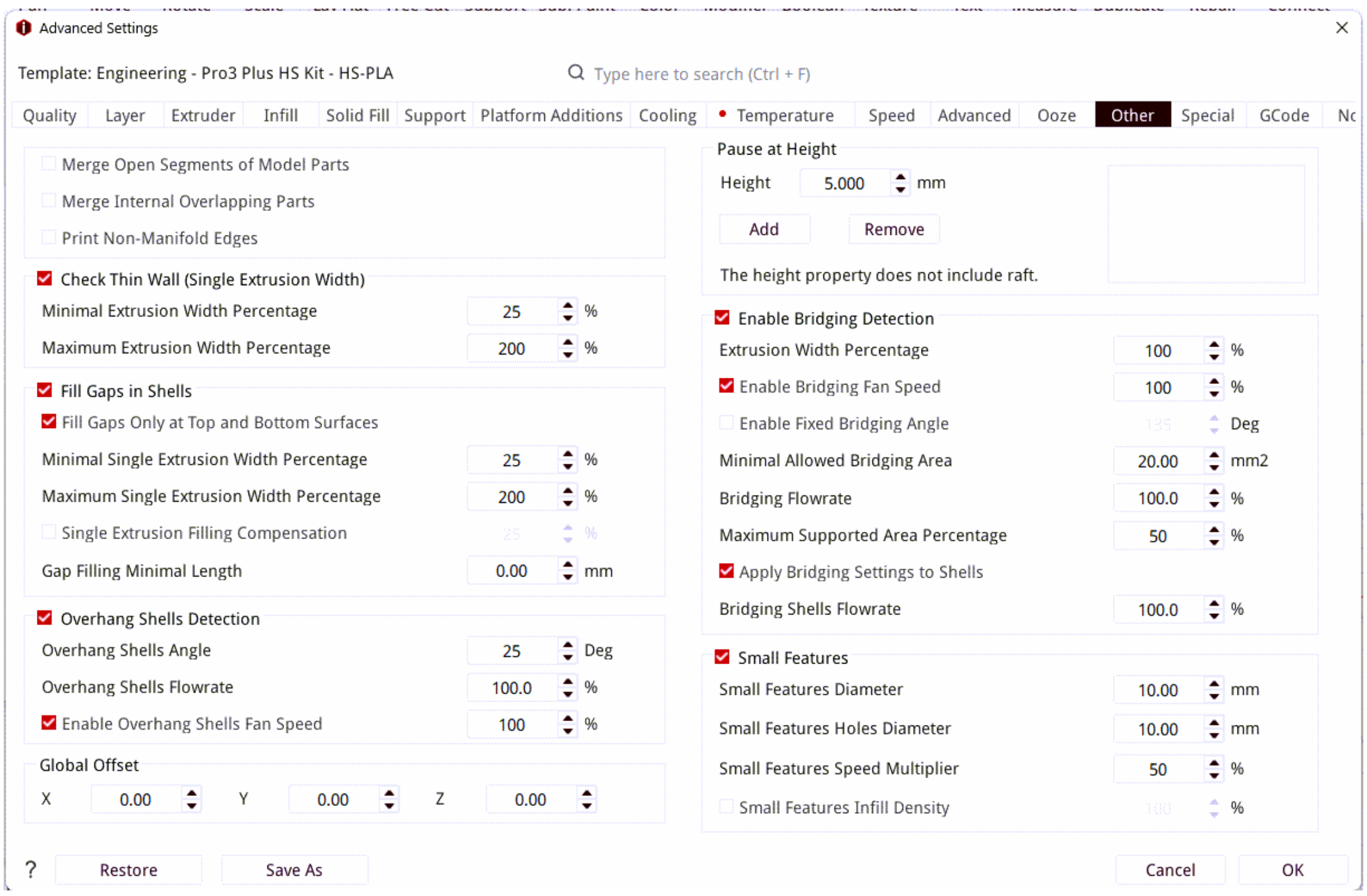
Advanced Settings
Template: Engineering - Pro3 Plus HS Kit - HS-PLA
Type here to search (Ctrl + F)
Quality
Layer
Extruder
Infill
Solid Fill
Support
Platform Additions
Cooling
Temperature
Speed
Advanced
Ooze
Other
Special
GCode
Merge Open Segments of Model Parts
Merge Internal Overlapping Parts
Print Non-Manifold Edges
Check Thin Wall (Single Extrusion Width)
Minimal Extrusion Width Percentage 25 %
Maximum Extrusion Width Percentage 200 %
Fill Gaps in Shells
Fill Gaps Only at Top and Bottom Surfaces
Minimal Single Extrusion Width Percentage 25 %
Maximum Single Extrusion Width Percentage 200 %
Single Extrusion Filling Compensation
Gap Filling Minimal Length 0.00 mm
Overhang Shells Detection
Overhang Shells Angle 25 Deg
Overhang Shells Flowrate 100.0 %
Enable Overhang Shells Fan Speed 100 %
Global Offset
X 0.00 Y 0.00 Z 0.00
Pause at Height
Height 5.000 mm
Add
Remove
The height property does not include raft.
Enable Bridging Detection
Extrusion Width Percentage 100 %
Enable Bridging Fan Speed 100 %
Enable Fixed Bridging Angle Deg
Minimal Allowed Bridging Area 20.00 mm2
Bridging Flowrate 100.0 %
Maximum Supported Area Percentage 50 %
Apply Bridging Settings to Shells
Bridging Shells Flowrate 100.0 %
Small Features
Small Features Diameter 10.00 mm
Small Features Holes Diameter 10.00 mm
Small Features Speed Multiplier 50 %
Small Features Infill Density %
?
Restore
Save As
Cancel
OK

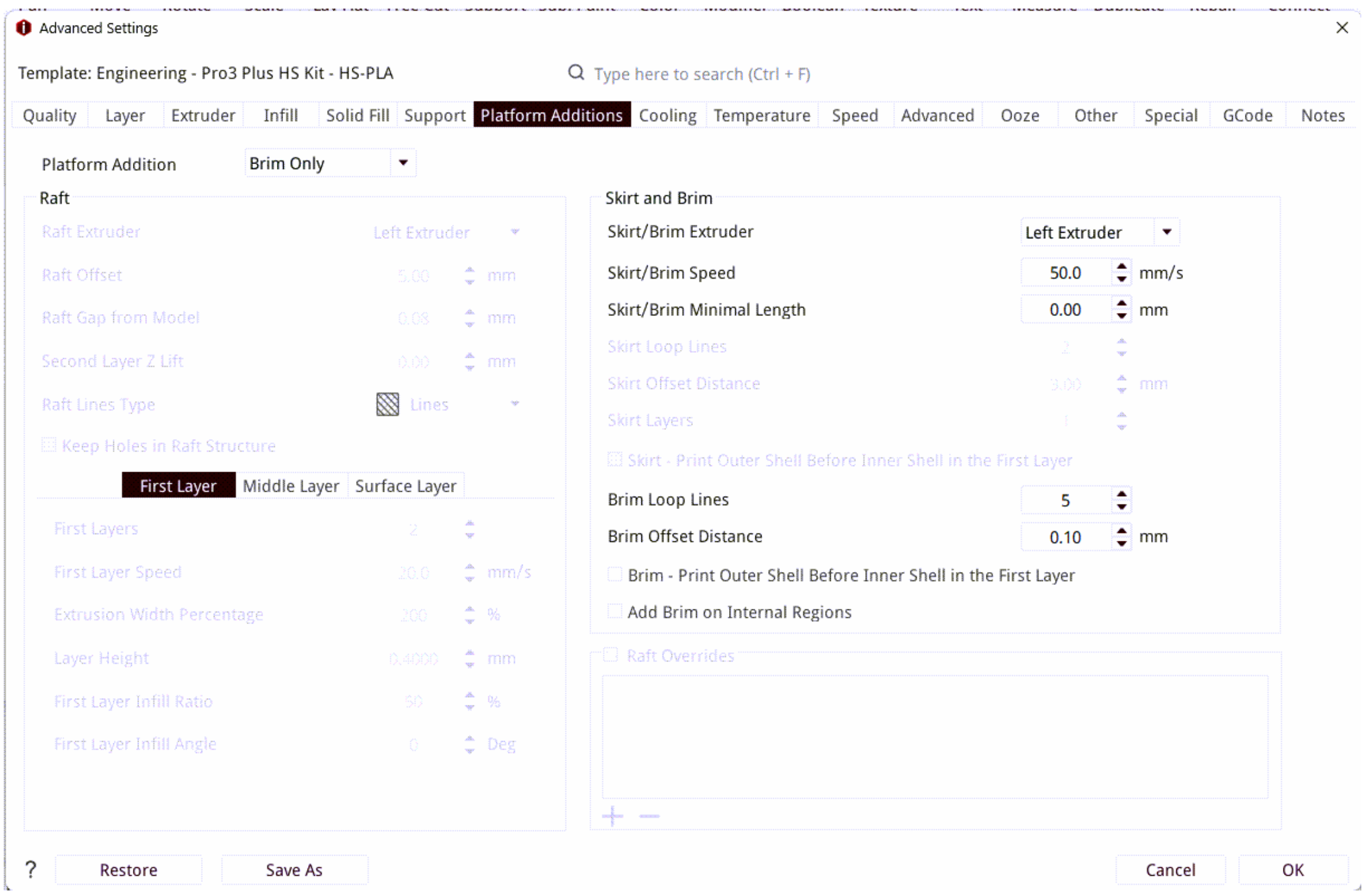
Advanced Settings
Template: Engineering - Pro3 Plus HS Kit - HS-PLA
Type here to search (Ctrl + F)
Quality Layer Extruder Infill Solid Fill Support Platform Additions Cooling Temperature Speed Advanced Ooze Other Special GCode Notes
Platform Addition Brim Only
Raft
Raft Extruder Left Extruder
Raft Offset mm
Raft Gap from Model mm
Second Layer Z Lift mm
Raft Lines Type Lines
Keep Holes in Raft Structure
First Layer Middle Layer Surface Layer
First Layers
First Layer Speed mm/s
Extrusion Width Percentage %
Layer Height mm
First Layer Infill Ratio %
First Layer Infill Angle Deg
Skirt and Brim
Skirt/Brim Extruder Left Extruder
Skirt/Brim Speed 50.0 mm/s
Skirt/Brim Minimal Length 0.00 mm
Skirt Loop Lines
Skirt Offset Distance mm
Skirt Layers
Skirt - Print Outer Shell Before Inner Shell in the First Layer
Brim Loop Lines 5
Brim Offset Distance 0.10 mm
Brim - Print Outer Shell Before Inner Shell in the First Layer
Add Brim on Internal Regions
Raft Overrides
?
Restore
Save As
Cancel
OK

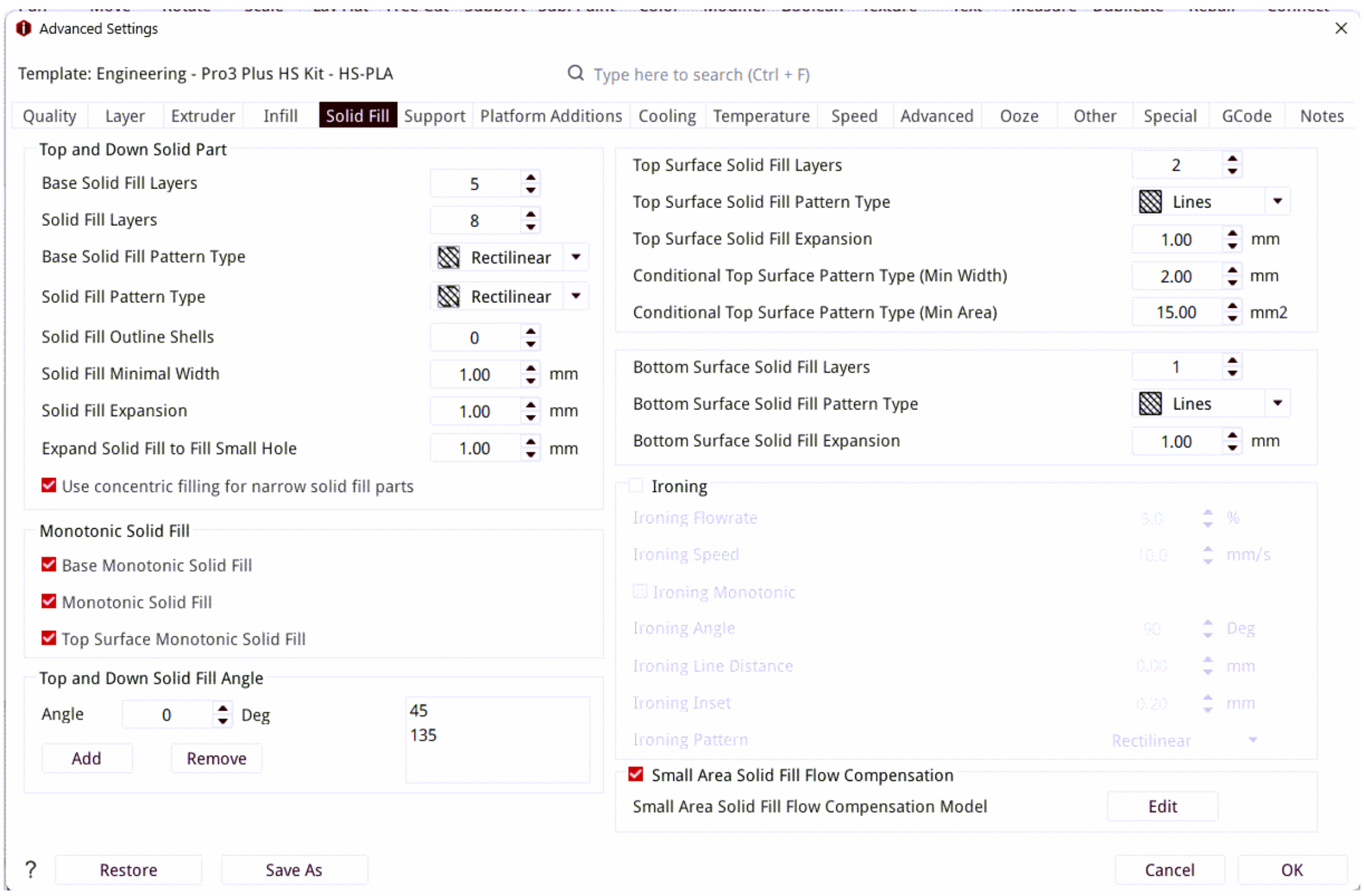
Advanced Settings
Template: Engineering - Pro3 Plus HS Kit - HS-PLA
Type here to search (Ctrl + F)
Quality Layer Extruder Infill Solid Fill Support Platform Additions Cooling Temperature Speed Advanced Ooze Other Special GCode Notes
Top and Down Solid Part
Base Solid Fill Layers 5
Solid Fill Layers 8
Base Solid Fill Pattern Type Rectilinear
Solid Fill Pattern Type Rectilinear
Solid Fill Outline Shells 0
Solid Fill Minimal Width 1.00 mm
Solid Fill Expansion 1.00 mm
Expand Solid Fill to Fill Small Hole 1.00 mm
Use concentric filling for narrow solid fill parts
Monotonic Solid Fill
Base Monotonic Solid Fill
Monotonic Solid Fill
Top Surface Monotonic Solid Fill
Top and Down Solid Fill Angle
Angle 0 Deg
45
135
Add
Remove
Top Surface Solid Fill Layers 2
Top Surface Solid Fill Pattern Type Lines
Top Surface Solid Fill Expansion 1.00 mm
Conditional Top Surface Pattern Type (Min Width) 2.00 mm
Conditional Top Surface Pattern Type (Min Area) 15.00 mm2
Bottom Surface Solid Fill Layers 1
Bottom Surface Solid Fill Pattern Type Lines
Bottom Surface Solid Fill Expansion 1.00 mm
Ironing
Ironing Flowrate %
Ironing Speed mm/s
Ironing Monotonic
Ironing Angle Deg
Ironing Line Distance mm
Ironing Inset mm
Ironing Pattern Rectilinear
Small Area Solid Fill Flow Compensation
Small Area Solid Fill Flow Compensation Model
Edit
?
Restore
Save As
Cancel
OK

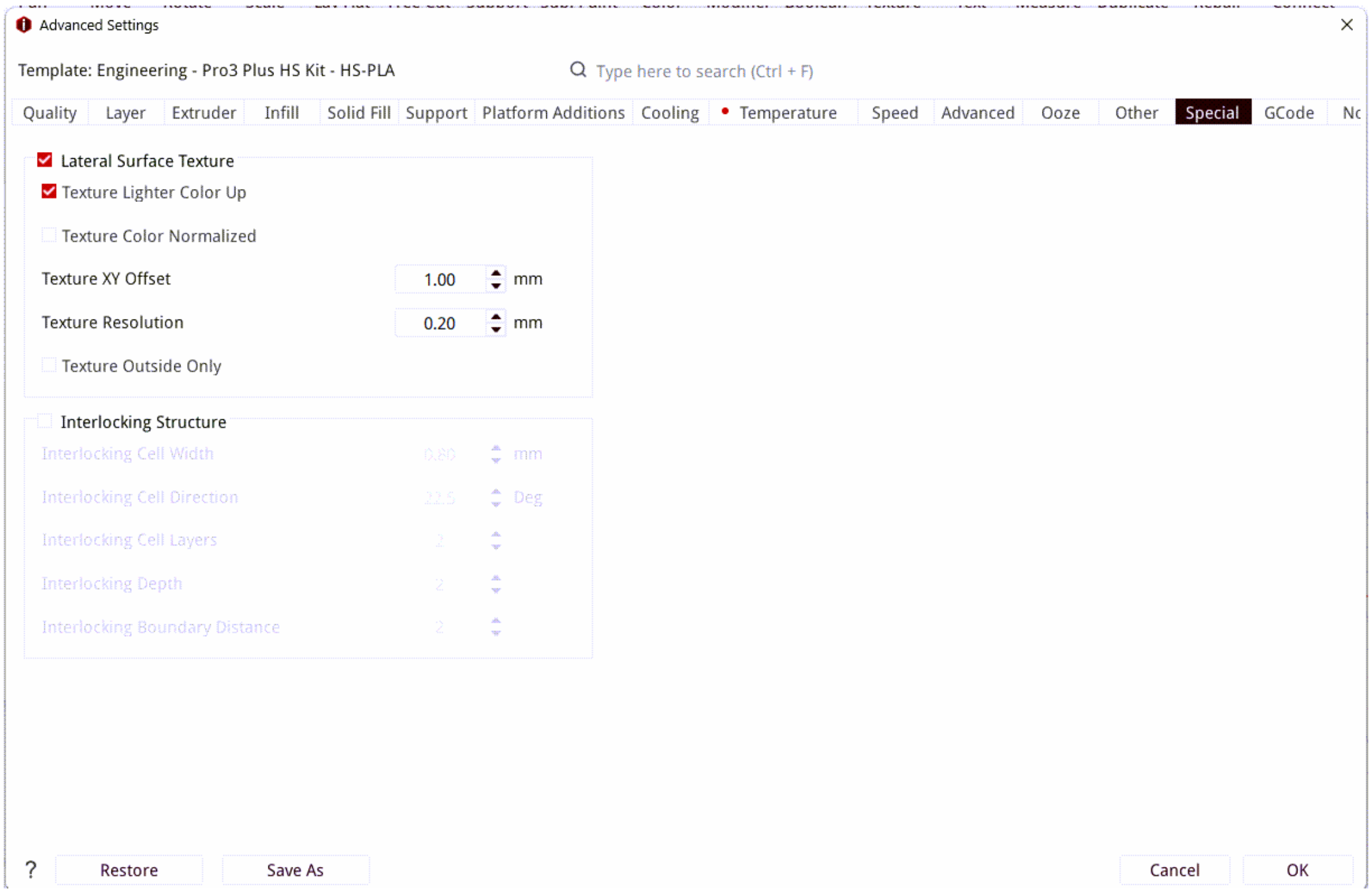
Advanced Settings
Template: Engineering - Pro3 Plus HS Kit - HS-PLA
Type here to search (Ctrl + F)
Quality
Layer
Extruder
Infill
Solid Fill
Support
Platform Additions
Cooling
Temperature
Speed
Advanced
Ooze
Other
Special
GCode
Lateral Surface Texture
Texture Lighter Color Up
Texture Color Normalized
Texture XY Offset 1.00 mm
Texture Resolution 0.20 mm
Texture Outside Only
Interlocking Structure
Interlocking Cell Width 0.80 mm
Interlocking Cell Direction 22.5 Deg
Interlocking Cell Layers 2
Interlocking Depth 2
Interlocking Boundary Distance 2
?
Restore
Save As
Cancel
OK

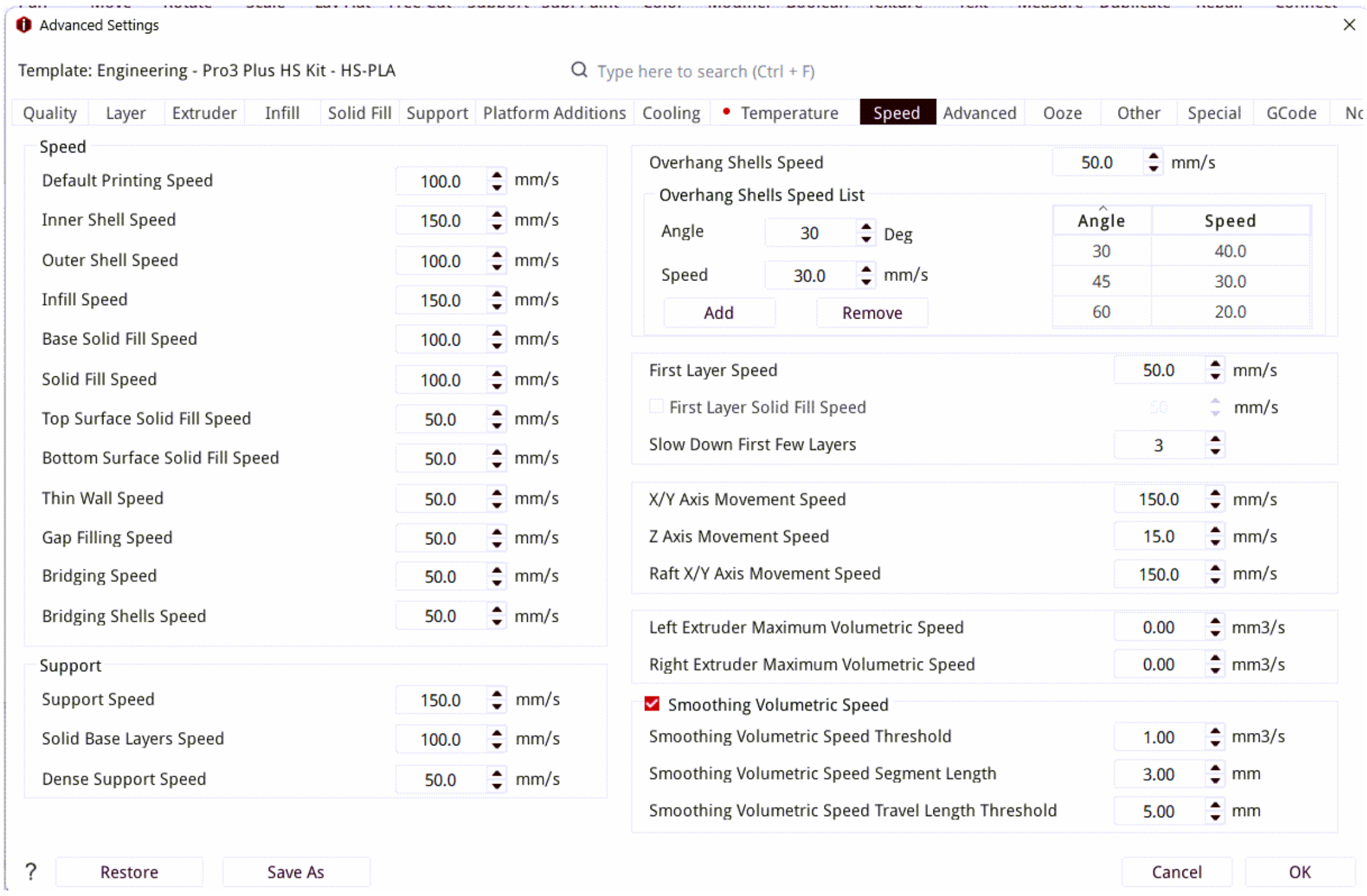
Advanced Settings
Template: Engineering - Pro3 Plus HS Kit - HS-PLA
Type here to search (Ctrl + F)
Quality
Layer
Extruder
Infill
Solid Fill
Support
Platform Additions
Cooling
Temperature
Speed
Advanced
Ooze
Other
Special
GCode
Speed
Default Printing Speed 100.0 mm/s
Inner Shell Speed 150.0 mm/s
Outer Shell Speed 100.0 mm/s
Infill Speed 150.0 mm/s
Base Solid Fill Speed 100.0 mm/s
Solid Fill Speed 100.0 mm/s
Top Surface Solid Fill Speed 50.0 mm/s
Bottom Surface Solid Fill Speed 50.0 mm/s
Thin Wall Speed 50.0 mm/s
Gap Filling Speed 50.0 mm/s
Bridging Speed 50.0 mm/s
Bridging Shells Speed 50.0 mm/s
Support
Support Speed 150.0 mm/s
Solid Base Layers Speed 100.0 mm/s
Dense Support Speed 50.0 mm/s
Overhang Shells Speed 50.0 mm/s
Overhang Shells Speed List
Angle 30 Deg
Speed 30.0 mm/s
Add
Remove
Angle Speed
30 40.0
45 30.0
60 20.0
First Layer Speed 50.0 mm/s
First Layer Solid Fill Speed 50 mm/s
Slow Down First Few Layers 3
X/Y Axis Movement Speed 150.0 mm/s
Z Axis Movement Speed 15.0 mm/s
Raft X/Y Axis Movement Speed 150.0 mm/s
Left Extruder Maximum Volumetric Speed 0.00 mm3/s
Right Extruder Maximum Volumetric Speed 0.00 mm3/s
Smoothing Volumetric Speed
Smoothing Volumetric Speed Threshold 1.00 mm3/s
Smoothing Volumetric Speed Segment Length 3.00 mm
Smoothing Volumetric Speed Travel Length Threshold 5.00 mm
?
Restore
Save As
Cancel
OK

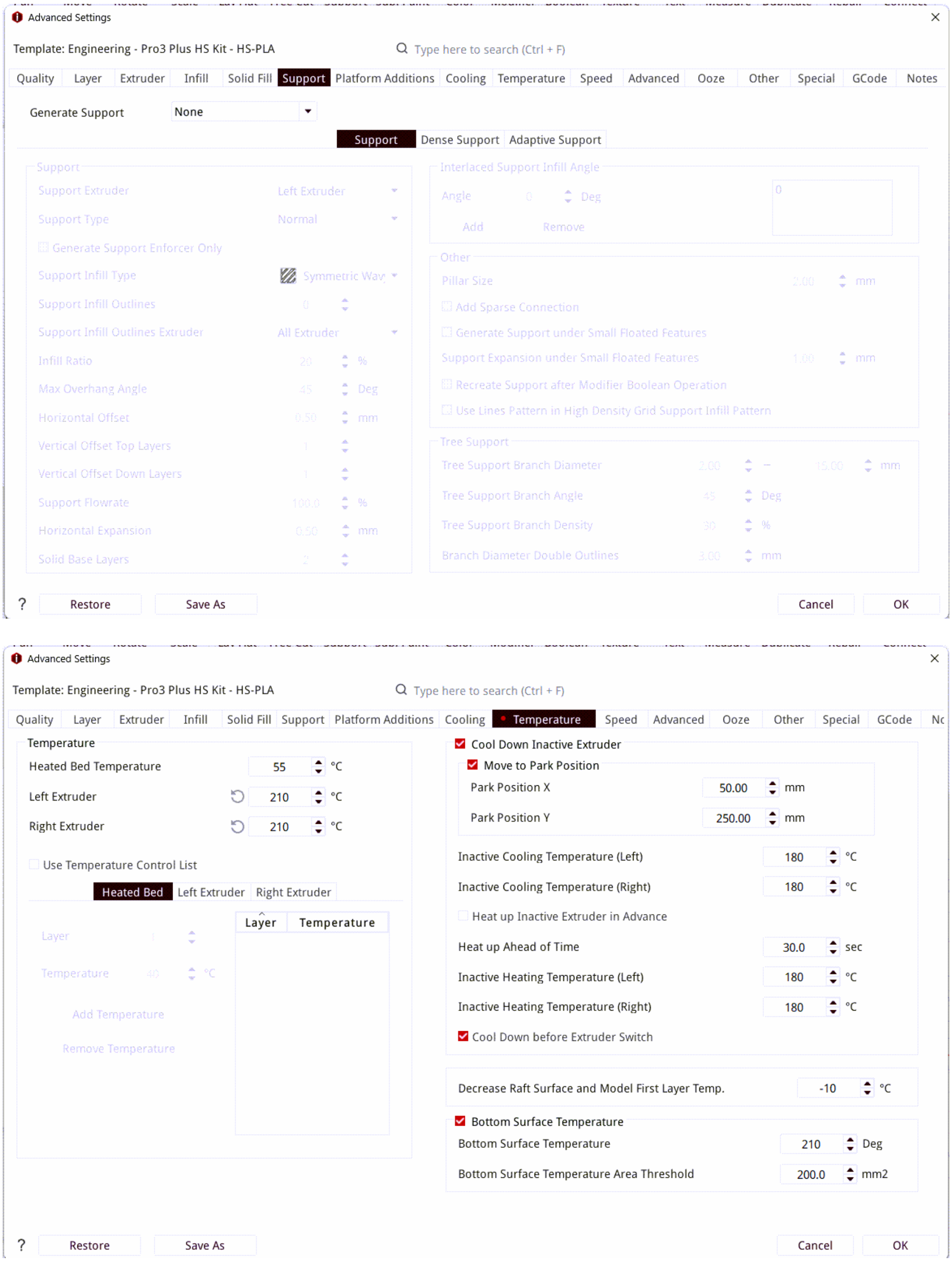

Figure S2-1. Native parameter interface of the ideaMaker slicing software (Raise3D)

S2.2 Print Orientation Strategy

Figure S2-2 shows the 3D preview interface of the slicing software, illustrating the standardized print orientation strategy applied across all parts: the layer plane is aligned parallel to the primary loading direction of the part (e.g., for button caps, layers are oriented perpendicular to the press axis; for cable clips, layers are aligned along the clip-opening axis). This orientation minimizes the impact of FDM interlayer anisotropy, consistent with the risk-based mechanical validation framework described in Section 3.4 of the main manuscript.

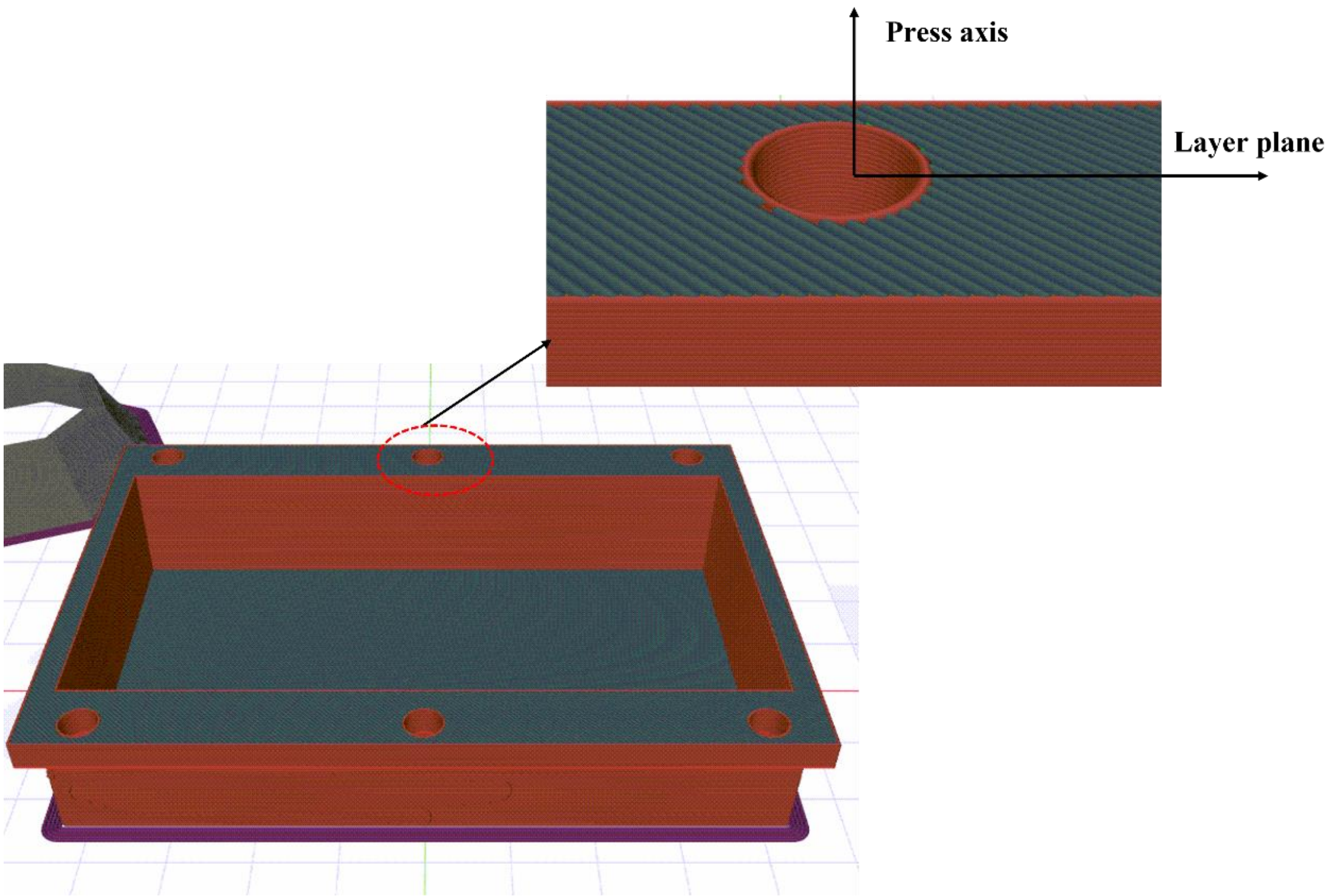

Figure S2-2 3D preview interface of the slicing software

S2.3 Iterative Adjustment Protocol

For the 6 initially unsuccessful prints described in Supplementary Material S1, the above baseline parameters were iteratively tuned within the ranges noted (e.g., bed temperature increased to 60°C for large flat parts, nozzle temperature increased to 215°C for high-stress snap-fits). All adjustments were documented in the hospital’s equipment maintenance log and are reflected in the failure analysis in S1.

All parameter configurations and slicing profiles referenced in this supplement are available from the corresponding author upon reasonable request, to support clinical engineering teams replicating our workflow.
